# Nutrigenomics in *Arma chinensis*: Transcriptome Analysis of *Arma chinensis* Fed on Artificial Diet and Chinese Oak Silk Moth *Antheraea pernyi* Pupae

**DOI:** 10.1371/journal.pone.0060881

**Published:** 2013-04-11

**Authors:** Deyu Zou, Thomas A. Coudron, Chenxi Liu, Lisheng Zhang, Mengqing Wang, Hongyin Chen

**Affiliations:** 1 Key Laboratory of Integrated Pest Management in Crops, Ministry of Agriculture, Institute of Plant Protection, Chinese Academy of Agricultural Sciences, Beijing, China; 2 Sino-American Biological Control Laboratory, USDA-Agricultural Research Service, Beijing, China; 3 Biological Control of Insects Research Laboratory, USDA-Agricultural Research Service, Columbia, Missouri, United States of America; Auburn University, United States of America

## Abstract

**Background:**

The insect predator, *Arma chinensis*, is capable of effectively controlling many pests, such as Colorado potato beetle, cotton bollworm, and mirid bugs. Our previous study demonstrated several life history parameters were diminished for *A. chinensis* reared on an artificial diet compared to a natural food source like the Chinese oak silk moth pupae. The molecular mechanisms underlying the nutritive impact of the artificial diet on *A. chinensis* health are unclear. So we utilized transcriptome information to better understand the impact of the artificial diet on *A. chinensis* at the molecular level.

**Methodology/Principal Findings:**

Illumina HiSeq2000 was used to sequence 4.79 and 4.70 Gb of the transcriptome from pupae-fed and artificial diet-fed *A. chinensis* libraries, respectively, and a *de novo* transcriptome assembly was performed (Trinity short read assembler). This resulted in 112,029 and 98,724 contigs, clustered into 54,083 and 54,169 unigenes for pupae-fed and diet-fed *A. chinensis,* respectively. Unigenes from each sample’s assembly underwent sequence splicing and redundancy removal to acquire non-redundant unigenes. We obtained 55,189 unigenes of *A. chinensis*, including 12,046 distinct clusters and 43,143 distinct singletons. Unigene sequences were aligned by BLASTx to nr, Swiss-Prot, KEGG and COG (E-value <10^−5^), and further aligned by BLASTn to nt (E-value <10^−5^), retrieving proteins of highest sequence similarity with the given unigenes along with their protein functional annotations. Totally, 22,964, 7,898, 18,069, 15,416, 8,066 and 5,341 unigenes were annotated in nr, nt, Swiss-Prot, KEGG, COG and GO, respectively. We compared gene expression variations and found thousands of genes were differentially expressed between pupae-fed and diet-fed *A. chinensis*.

**Conclusions/Significance:**

Our study provides abundant genomic data and offers comprehensive sequence information for studying *A. chinensis*. Additionally, the physiological roles of the differentially expressed genes enable us to predict effects of some dietary ingredients and subsequently propose formulation improvements to artificial diets.

## Introduction

*Arma chinensis* is a predaceous insect species that preys upon a large variety of species, and can effectively suppress agricultural and forest pests in the orders Lepidoptera, Coleoptera, Hymenoptera and Hemiptera [1]–[8]. Colorado potato beetle, *Leptinotarsa decemlineata*, one of the most important coleopteran exotic pests in Asia, has developed resistance to most insecticides used for its control [9], and has the potential to develop resistance to *Bacillus thuringiensis* (Bt) toxins [10], [11]. The application of transgenic Bt crops has suppressed lepidopteran insects such as the cotton bollworm *Helicoverpa armigera*. However, Lu *et al*. concluded that mirid bugs (Heteroptera: Miridae) in northern China have progressively increased in population size and acquired pest status in cotton and multiple other crops, in association with a regional increase in Bt cotton adoption [12]. Thus, the release of *A. chinensis* in association with transgenic crops may be a sustainable biocontrol strategy to decrease dependence on insecticides.

Mass rearing of biocontrol insects is important given the environmental, health and resistance issues associated with the use of chemical insecticides [13]. To achieve an effective level of control, however, requires a large number of beneficial insects be available at low cost for their augmentative and inoculative release. Conventional rearing methods require raising natural prey and (or) host insects on host plants. The extensive input necessary for this method makes the use of beneficial insects economically unfeasible. However, the development of artificial diets could considerably reduce the mass propagation costs of beneficial insects [14]–[18]. An insect-free artificial diet comprised of pig liver and tuna was developed for *A. chinensis*. Fecundity and egg viability were lower for diet-fed *A. chinensis* compared to *A. chinensis* reared on pupae of the natural prey, the Chinese oak silk moth *Antheraea pernyi*. Developmental time from 2nd instar to adult and the preovipositional period were significantly longer for diet-fed *A. chinensis*. Nymphal weight, body length, adult longevity, survival from 2nd instar to adult, and fertility increased, while sex ratio (♀/♂) decreased, with the rearing of consecutive generations on the diet. In particular, the longevity of adults reared on the artificial diet was significantly longer than of those reared on pupae. Additionally, the diet-fed *A. Chinensis* released less defensive odor than did the pupae-fed insects.

The current method for optimizing diet is to measure a few pre-selected biochemical and (or) physiological parameters to test the effect of changes in diet formulation on insect performance [18]–[21]. Typically, diet components are changed one at a time and insect performance is tested after each change. This endeavor is time-consuming, taking years to decades to optimize a diet, with many attempts ending in failure. To accelerate diet development, a more direct method that can provide informative feedback to target deficiencies in diet formulation is required. Nutrigenomics examines how nutrition affects gene expression patterns and offers not only a means to measure an insect’s response to changes in the food stream but also provides information on diet limitations [22]. Using suppressive subtractive hybridization, Yocum *et al.* discovered two artificial diet up-regulated and two prey up-regulated transcript fragments in the predatory pentatomid *Perillus bioculatus*, and a BLASTx search found similarities for two diet up-regulated clones, i.e., the tyrosine-3-monooxygenase gene and the chitin binding protein gene, Gasp [22]. Coudron *et al.* reared groups of oriental fruit fly, *Bactrocera dorsalis* separately on media either devoid of, or supplemented with, wheat germ oil and identified one gene encoding receptor for activated C kinase 1 that increased in expression by 6.8-fold in eggs from adults reared on media supplemented with wheat germ oil. The receptor for activated C kinase 1 is an essential component of at least three intracellular signal transduction pathways, making it a good candidate molecular marker of lipid deficiency in fruit flies and possibly many other insect species [23]. Alaux *et al.* compared the transcriptome of bees fed with pollen and sugar and bees restricted to a sugar diet, and found that pollen activated nutrient-sensing and metabolic pathways. In addition, those nutrients had a positive influence on genes affecting longevity and the production of some antimicrobial peptides [24]. Coudron *et al.* found that trace element levels in *Podisus maculiventris* were substantially affected by food source and could vary significantly from levels in the food source [25]. These studies demonstrated the feasibility of using nutrigenomics to assist in analyzing insect responses to nutritional changes and dietary quality with the intent of improving insect diets.

Currently, next generation high-throughput sequencing techniques (Solexa/Illumina, Roche 454) provide a unique opportunity for genomic exploration in insect species where little or no molecular knowledge is available [26]. This technology greatly increases the quantity of data that can be generated in a short time at a reduced cost [27]. For example, Illumina sequencing has been applied in *Nilaparvata lugens* [28], *Apis mellifera* [24], *Bemisia tabaci* [29], [30], *Sogatella furcifera* [31], *B. dorsalis* [32] and *Pogonus chalceus* [33] research. The use of 454-sequencing technology has enabled the application of functional genomics to a broad range of insect species including *Cimex lectularius* [34], *Melitaea cinxia* [35], *Zygaena filipendulae* [36], *Chyrsomela tremulae* [37], *Aphis glycines* [38], *Manduca sexta* [39], [40], *Laodelphax striatellus* [41], *Stomoxys calcitrans* [42], *Dermacentor variabilis* [43], *Erynnis properties* and *Papilio zelicaon* [44] and *Agrilus planipennis* [45]. To date, however, transcriptome analysis has not yet been utilized to assist in artificial diet formulation for a beneficial predatory insect. In this study, we applied Illumina sequencing technology to obtain the transcriptome of *A. chinensis* and compared the differentially expressed genes between *A. chinensis* fed with Chinese oak silk moth pupae and those fed with an artificial diet. Our results will allow for a better understanding of the food-driven molecular processes in *A. chinensis* and the identification of potentially useful candidate genes in gene-assisted diet formulation.

## Results and Discussion

### Sequencing and Sequence Assembly

After removal of adaptor sequences, ambiguous reads and low-quality reads (Q20<20), a total of 53,224,704 (SRA accession number SRR617645) and 52,244,538 (SRA accession number SRR618073) high-quality clean reads comprised of 4,790,223,360 nucleotides (4.79 Gb) and 4,702,008,420 nucleotides (4.70 Gb) from the Chinese oak silk moth pupae-fed (CY_1) and artificial diet-fed (AD_1) *A. chinensis* libraries were generated, respectively. All high-quality reads were assembled *de novo* into 112,029 (CY_1) and 98,724 (AD_1) contigs using the Trinity program [46], with an N50 of 318 bp (CY_1) and 494 bp (AD_1) (i.e. 50% of assembled bases were incorporated into contigs of 318 bp and 494 bp (or longer) for pupae-fed and diet-fed insects (Table S1).

A total of 112,029 contigs of CY_1 were assembled, with a total length of 28,024,077 nt and mean length of 250 bp. Similarly, a total of 98,724 contigs of AD_1 were assembled, with a total length of 30,459,937 nt and mean length of 309 bp (Table S1). Size distribution indicated that the lengths of the 4,578 and 6,773 contigs were more than 1000 bp for CY_1 and AD_1, respectively (Figure S1C and S1A). Using paired-end reads and gap-filling, these contigs were further assembled and clustered into unigenes. Finally, we obtained 54,083 and 54,169 unigenes of CY_1 and AD_1, including 8,636 and 10,315 distinct clusters and 45,447 and 43,854 distinct singletons with a mean length of 427 and 541 bp, respectively (Table S1). Among these unigenes, the lengths of the 5,334 CY_1 unigenes and 8,681 AD_1 unigenes were more than 1000 bp (Figure S1D and S1B). Unigenes from each sample assembly underwent sequence splicing and redundancy removal to acquire longer non-redundant All-unigenes, given these two samples were sequenced from the same species.

### Summary of Annotation Results

Functional annotation of unigenes includes information on proteins, COG, and Gene Ontology (GO). Unigene sequences were first aligned by BLASTx to protein databases nr, Swiss-Prot, KEGG and COG (E-value <10^−5^), and then aligned by BLASTn to nucleotide databases nt (E-value <10^−5^), retrieving proteins of highest sequence similarity with the given unigenes and their protein functional annotations.

We obtained 55,189 unigenes of *A. chinensis* from both food treatments, including 12,046 distinct clusters and 43,143 distinct singletons with a mean length of 583 bp (Table S1). Size distribution indicated that the lengths of the 9,262 unigenes were more than 1000 bp (Figure S2A). Of those, 22,964 (41.61%), 7,898 (14.31%), 18,069 (32.74%), 15,416 (27.93%), 8,066 (14.62%) and 5,341 (9.68%) unigenes were annotated in nr, nt, Swiss-Prot, KEGG, COG and GO, respectively. A total of 24,187 unigenes were annotated in one or more of the databases (43.83% of all unigenes), suggesting they have relatively well conserved functions.

### Annotation of Predicted Proteins

For functional annotation, distinct gene sequences were searched using BLASTx against nr NCBI nucleotide database with a cut-off E-value of 10^−5^. A total of 22,964 genes returned an above cut-off BLAST result, representing 41.61% of all distinct sequences. The E-value distribution of the top hits in the nr database showed that 39.2% of the mapped sequences had strong homology (smaller than 1.0E^−45^), whereas 60.7% of the homolog sequences ranged between 1.0E^−5^ to 1.0E^−45^ (Figure S3A). Likewise, similarity distribution showed that 29.3% of the sequences had a similarity higher than 60%, while 70.7% had a similarity ranging from 16% to 60% (Figure S3B). The highest percentage of *A. chinensis* sequences were matched to *Tribolium castaneum* (14.8%), followed by *Acyrthosiphon pisum* (11.5%), *Nasonia vitripennis* (6.2%), *Camponotus floridanus* (5.4%), *Acromyrmex echinatior* (5.1%), *Harpegnathos saltator* (4.9%) and *Bombus impatiens* (4.4%) (Figure S3C).

### GO Assignments

**Figure 1:**
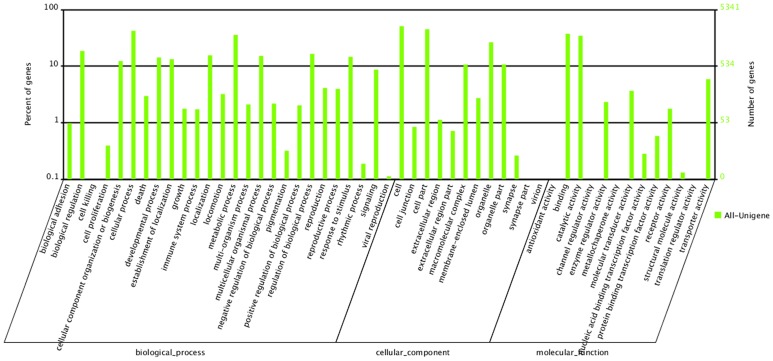
Gene Ontology (GO) categories of the unigenes. Unigenes were annotated in three categories: biological processes (26 sub-categories, 12,428 sequences), cellular components (12 sub-categories, 7,977 sequences), and molecular functions (13 sub-categories, 4,625 sequences).

In total 5,341 transcripts of *A. chinensis* were assigned to GO terms based on BLAST matches with previously known sequences (Figure 1, Table S2). These transcripts were associated with biological processes (26 sub-categories, 12,428 sequences), cellular components (12 sub-categories, 7,977 sequences) and molecular functions (13 sub-categories, 4,625 sequences). Among the molecular function assignments, a high percentage of genes were associated with binding functions (1995, 43.14%), predominantly heat shock proteins (Hsp) and catalytic activity (1844, 39.87%). In a recent study of *C. lectularius*, the transcript levels for Hsp 70 and Hsp 90 were elevated when bugs were subjected to various stress factors (heat, cold and dehydration) suggesting that these proteins may play an important role during environmental stress and could potentially play a role in control strategies [47]–[50]. Cellular component sequences in the present study showed a significant percentage of genes assigned to cellular (2720, 34.10%) and cell component (2397, 30.05%) functions, whereas biological process sequences were associated predominantly with cellular processes (2254, 18.14%) such as proteolysis, carbohydrate metabolic processes and oxidation reduction utilization, and metabolic processes (1906, 15.34%). Similar observations for metabolic processes have been reported in transcriptomic studies of other insects [28], [29], [32], [34], [40], [45].

### COG Classification

**Figure 2:**
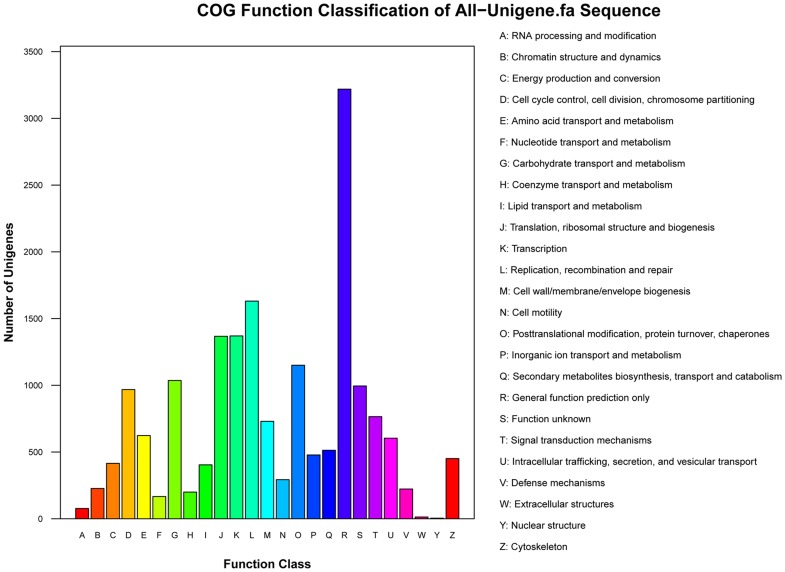
Clusters of Orthologous Groups (COG) functional classification. From 22,964 nr hits, 8,066 sequences were classified into 25 COG categories (E-value <1.0^−5^).

To further evaluate the completeness of our transcriptome library and the effectiveness of our annotation process, we searched the annotated sequences for genes involved in COG classifications. From 22,964 nr hits, 8,066 sequences had a COG classification (Figure 2). Among the 25 COG categories, the cluster for ‘General function prediction only’ represented the largest group (3,219, 39.91%) followed by ‘Replication, recombination and repair’ (1,631, 20.22%), ‘Transcription’ (1,370, 16.98%), ‘Translation, ribosomal structure and biogenesis’ (1,367, 16.95%) and ‘Posttranslational modification, protein turnover, chaperones’ (1,150, 14.26%). Nuclear structure (4, 0.05%), extracellular structures (13, 0.16%) and RNA processing and modification (77, 0.95%) represented the smallest groups (Figure 2).

### KEGG Analysis

To identify active biological pathways in *A. chinensis*, we mapped the 22,964 annotated sequences to the reference canonical pathways in KEGG [51]. In total, we assigned 15,416 sequences to 240 KEGG pathways. The pathways most represented by the unique sequences were metabolic pathways (2,333, 15.13%), regulation of actin cytoskeleton (568, 3.68%), focal adhesion (543, 3.52%), pathways in cancer (540, 3.5%) and purine metabolism (473, 3.07%). The smallest groups were lysine biosynthesis (3, 0.02%), asthma (3, 0.02%), polyketide sugar unit biosynthesis (1, 0.01%) and intestinal immune network for Immunoglobulin A (IgA) production (1, 0.01%) (Table S3). These annotations provide a valuable resource for investigating specific processes, functions, and pathways during nutrigenomics research of *A. chinensis*.

### Protein Coding Region Prediction

Unigenes were first aligned by BLASTx (E-value <10^−5^) to protein databases in the priority order of nr, Swiss-Prot, KEGG, and COG. Unigenes that aligned to a higher priority database were not aligned to a lower priority database. Proteins with the highest BLAST ranks were selected to decide the coding region sequences of unigenes, which were then translated into amino acid sequences with the standard codon table. Both nucleotide (5′–3′) and amino acid sequences of the unigene coding region were acquired. We obtained a total of 23,124 significant BLAST hits (41.90% of all unigenes). The size distribution for the protein coding sequences (CDS) and predicted proteins are shown in Figure S2B and S2D. Unigenes not aligned to any database were scanned by ESTScan [52], producing nucleotide sequence (5′–3′) directions and amino acid sequences of the predicted coding region. A total of 4,292 unigenes were analyzed using ESTScan (size distributions of the ESTs and proteins are shown in Figure S2C and S2E).

### Differentially Expressed Genes (DEG) Statistics

**Figure 3:**
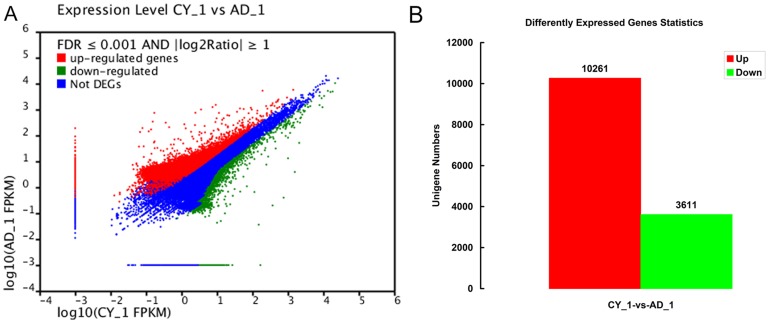
Expression level and statistics of the differentially expressed genes (DEG) in artificial diet-fed (AD_1) vs. prey-fed (CY_1) treatments. (A) Expression level of DEG. (B) Statistics of DGE. In total, the expression levels of 54,977 genes were affected by the artificial diet. Of which, 13,872 DEGs had significantly differential expression levels (FDR ≤ 0.001 and |log_2_Ratio| ≥ 1). Among them, 10,261 and 3,611 genes were up-regulated and down-regulated, respectively in AD_1 vs. CY_1.

The expression level of 54,977 genes were affected by the artificial diet, in which, 13,872 DEGs had significant differentially expressed levels (FDR ≤ 0.001 and |log_2_Ratio| ≥ 1) between the two food treatments. Among those, 10,261 were up-regulated and 3,611 were down-regulated in artificial diet-fed vs. prey-fed *A. chinensis* (Figure 3). Of the 30 most differentially up-regulated genes, 12 had defined functions, i.e., six energy metabolic related genes (*ATP synthase F0 subunit 6*, *NADH dehydrogenase subunit 1*, *2*, *3*, *4*, and *6*), two mitochondrial genes (*cytochrome c oxidase subunit I* and *cytochrome oxidase subunit II*), a structural cuticular protein (*apidermin 1 precursor*), a transmembrane receptor (*tollo*), *kinesin heavy chain* and *alpha 1 S haptoglobin*. Of the 30 most differentially down-regulated genes, 13 had defined functions, i.e., one energy metabolic related gene (*ATP synthase F0 subunit 6*), two mitochondrial genes (*cytochrome b* and *cytochrome oxidase subunit II*), three ribosome genes (*ribosomal protein l3*, *40S ribosomal protein S27*, and *60S ribosomal protein L6*), one translation initiation factor 2 (*PvLEA1 protein*), one globular protein (*beta tululin*), one RNA transport gene (*elongation factor 1-alpha*, Unigene41798_All), one coactivator (*MBF2*), two serine protease inhibitors (*pacifastin light chain precursor* and *serine protease inhibitor 28*), and one seminal fluid protein (*seminal fluid protein CSSFP066*). A total of 35 genes among the 60 differentially expressed genes had unknown functions or no annotations (Table S4). These top 30 most differentially up- and down-regulated genes demonstrated that the nutritional differences represented by Chinese oak silk moth pupae and artificial diet caused changes in a broad range of genes, yet both supported complete development of *A. chinensis.*

### GO Assignments of DEG

**Figure 4:**
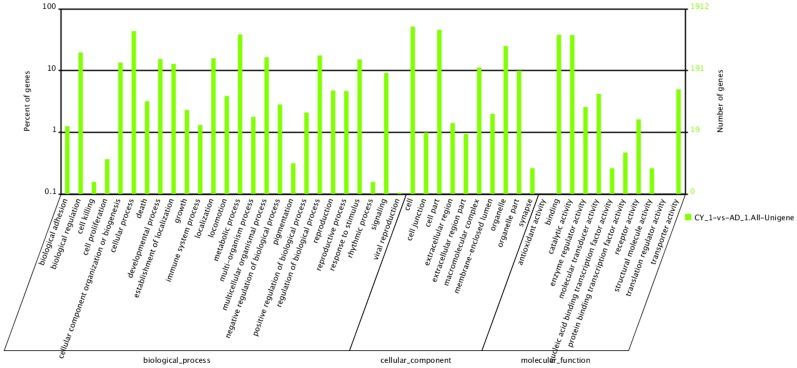
Gene Ontology (GO) categories of the differentially expressed genes (DEG) in artificial diet-fed (AD_1) vs. prey-fed (CY_1) treatments. In total, 1,912 DEGs of *A. chinensis* were annotated in three categories: biological processes (26 sub-categories, 4,713 sequences), cellular components (10 sub-categories, 2,853 sequences) and molecular functions (11 sub-categories, 1,725 sequences).

In total, 1,912 DEGs of *A. chinensis* were assigned to GO terms based on BLAST matches with sequences of known function (Figure 4, Table S5). These transcripts were assigned to biological processes (26 sub-categories, 4,713 sequences), cellular components (10 sub-categories, 2,853 sequences) and molecular functions (11 sub-categories, 1,725 sequences). Among the biological process terms, a high percentage of genes were assigned to cellular (832, 17.65%) and metabolic (735, 15.60%) processes. The cellular component terms showed a significant percentage of genes assigned to cells (987, 34.60%) and cell parts (875, 30.67%), whereas molecular function assignments were predominantly associated with binding (728, 42.20%) and catalytic activity (721, 41.80%).

### KEGG of DEG

To identify the biological pathways associated with genes differentially expressed between the food treatments, we mapped the 13,872 DEGs (FDR ≤ 0.001 and |log_2_Ratio| ≥ 1) and annotated sequences to the reference canonical pathways in KEGG [51]. In total, we assigned 5,879 sequences to 239 KEGG pathways. The pathways most represented by the DEGs were metabolic pathways (891, 15.16%), pathways in cancer (215, 3.66%), regulation of actin cytoskeleton (201, 3.42%), focal adhesion (178, 3.03%), influenza A (166, 2.82%), RNA transport (157, 2.67%), endocytosis (156, 2.65%), protein processing in endoplasmic reticulum (150, 2.55%), ubiquitin mediated proteolysis (150, 2.55%) and neuroactive ligand-receptor interaction (144, 2.45%). The smallest groups were intestinal immune network for IgA production (1, 0.02%), D-glutamine and D-glutamate metabolism (1, 0.02%), lysine biosynthesis (1, 0.02%), asthma (1, 0.02%), lipoic acid metabolism (2, 0.03%), D-arginine and D-ornithine metabolism (3, 0.05%), primary immunodeficiency (3, 0.05%), phenylalanine, tyrosine and tryptophan biosynthesis (3, 0.05%), thiamine metabolism (3, 0.05%) and biotin metabolism (4, 0.07%). These annotations provide a valuable resource for investigating specific processes, functions and pathways during nutrigenomics research of *A. chinensis*.

### DEG Related to Different Biological Characteristics

Several biological characteristics differed between pupae-fed and diet-fed *A. chinensis*, such as reduced fecundity, lower egg viability, prolonged nymphal development time, longer lifespan and higher cannibalism. We found several DEGs (FDR ≤ 0.001 and |log_2_Ratio| ≥ 2) related to these biological characteristics (Table S6). For reduced fecundity, three differentially expressed genes between the food treatments, specifically two *heat shock proteins 83-1* and *90*, associated with progesterone-mediated oocyte maturation, were markedly down-regulated in diet-fed insects.

In insects, seminal fluid proteins (Sfps) produced in the male accessory glands significantly increase male fitness by promoting sperm storage, temporarily increasing female egg-laying rate, and decreasing female sexual receptivity [53], thus increasing progeny production and delaying sperm displacement/competition [54]. Post-copulatory sexual selection can select for sperm allocation strategies in males [55] but males can also strategically allocate non-sperm components of the ejaculate [56], [57] such as Sfps. Thus, Sfps can influence the extent of post-copulatory sexual selection [58]–[60]. Using *Drosophila melanogaster*, Wigby *et al.* demonstrated that Sfps were strategically allocated to females in response to the potential level of sperm competition [55]. Males who were able to produce and transfer larger quantities of specific Sfps had a significant competitive advantage. Large male accessory glands also significantly increased competitive reproductive success. Quantitative variation in specific Sfps may play an important role in post-copulatory sexual selection and investment in Sfp production is essential for male fitness in a competitive environment [55]. Thus, the down-regulated *seminal fluid protein CSSFP066* in the artificial diet-fed *A. chinensis* could also contribute to reduced fecundity, lower egg viability, and a sex ratio shift in favor of males.

Among the DEGs for insect hormone biosynthesis, the gene *cytochrome P450 302a1* (ko: ecdysteroid 22-hydroxylase) (molting hormone) was up-regulated, as were juvenile hormone genes, *esterase FE4-like* (ko: juvenile-hormone esterase), *esterase FE4-like isoform 1* (ko: juvenile-hormone esterase), *beta-esterase 2 precursor* (ko: juvenile-hormone esterase), *venom carboxylesterase-6-like* (ko: juvenile-hormone esterase) and *pheromone-degrading enzyme 2* (ko: juvenile-hormone esterase). These up-regulated juvenile hormone genes may have contributed to the observed prolonged nymphal development time. Since the nutrition of the artificial diet increased longevity in *A. chinensis*, we looked at *Drosophila* data for insight on the expression pattern of genes found to affect longevity, as discovered by functional analysis from DAVID 6.7 bioinformatic resources [61] and GoToolbox [62]. Collectively, 20 genes known to effect longevity [24] were found, of which three were up-regulated in diet-fed *A. chinensis*, *superoxide dismutase [Cu-Zn]-like precursor* (Swiss-Prot: superoxide dismutase [Cu-Zn]) and *Cu, Zn-superoxide dismutase* encoding a cytoplasmic Cu-Zn superoxide dismutase, which increase lifespan [63], [64]. *Atpalpha* (*sodium pump alpha subunit*) with catalytic function was also up-regulated in diet-fed insects. Additionally, four DEGs, *antennal esterase CXE19*, *sensory appendage protein 1*, *defensin-like protein precursor* and *odorant binding protein 15,* related to higher cannibalism, were all down-regulated in the diet-fed insects, suggesting sensory ability to odors was possibly linked to the observed reduction in defense odors.

### KEGG of DEG Related to Artificial Diets

Honeybees require ten essential amino acids in their diet for their adult development: arginine, histidine, lysine, tryptophan, phenylalanine, methionine, threonine, leucine, isoleucine and valine [65]. Less is known about essential amino acids in *A. chinensis*. However, there was a trend in the diet-fed *A. chinensis* of up-regulation of sequences associated with the metabolism of all ten of the amino acids essential to the honey bee. Of those, DEGs enriched at a significant level (Q <0.05) only in one pathway (alanine, aspartate and glutamate metabolism) (Table S7).

Most DEGs enriched in the seven pathways related to fat metabolism were up-regulated in diet-fed insects, including adipocytokine signaling pathway, pyruvate metabolism, fatty acid biosynthesis, glycerolipid metabolism, fat digestion and absorption, fatty acid metabolism and fatty acid elongation (Table S7). This indicated that the artificial diet may contain excess lipids, suggesting that dietary reductions in tuna, chicken egg and pig liver were required. Most DEGs enriched in the four pathways related to starch and sugar metabolism were up-regulated in diet-fed insects, including carbohydrate digestion and absorption, and fructose and mannose metabolism (Table S7). Because sucrose was the main source of sugar, sucrose reductions were likely required in the diet. Most DEGs enriched in the ten metabolic pathways related to vitamins were up-regulated in diet-fed insects, including ascorbate and aldarate metabolism, vitamin digestion and absorption, folate biosynthesis, pantothenate and coenzyme A (CoA) biosynthesis, nicotinate and nicotinamide metabolism, biotin metabolism, retinol metabolism, thiamine metabolism, vitamin B6 metabolism and riboflavin metabolism (Table S7). Because vitamins, especially vitamin B, were added to the diet separately, the concentration of each vitamin in the diet could be reduced independently.

Zinke *et al.* categorized differentially expressed nutrient-controlled genes in *Drosophila* larvae into groups reflecting distinct physiological pathways mediating sugar metabolism such as lipase 3, glucose transporter, insulin receptor, as well as fatty acid synthase, fat metabolism such as acetyl CoA carboxylase, acyl CoA thioesterhydrolase, ATP-citrate lyase, *Zwischenferment*, glucose-6-phosphate dehydrogenase, and triacylglycerol lipases [66]. For diet-fed *A. chinensis*, we also found some differentially expressed nutrient-controlled genes, including sugar-lipase-3, glucose transporter, insulin receptor, fatty acid synthase and acetyl CoA carboxylase, which were all up-regulated in the diet-fed insects (Table S8). These differentially expressed nutrient-controlled genes again demonstrated that fat and sugar should be reduced in the artificial diet.

**Figure 5:**
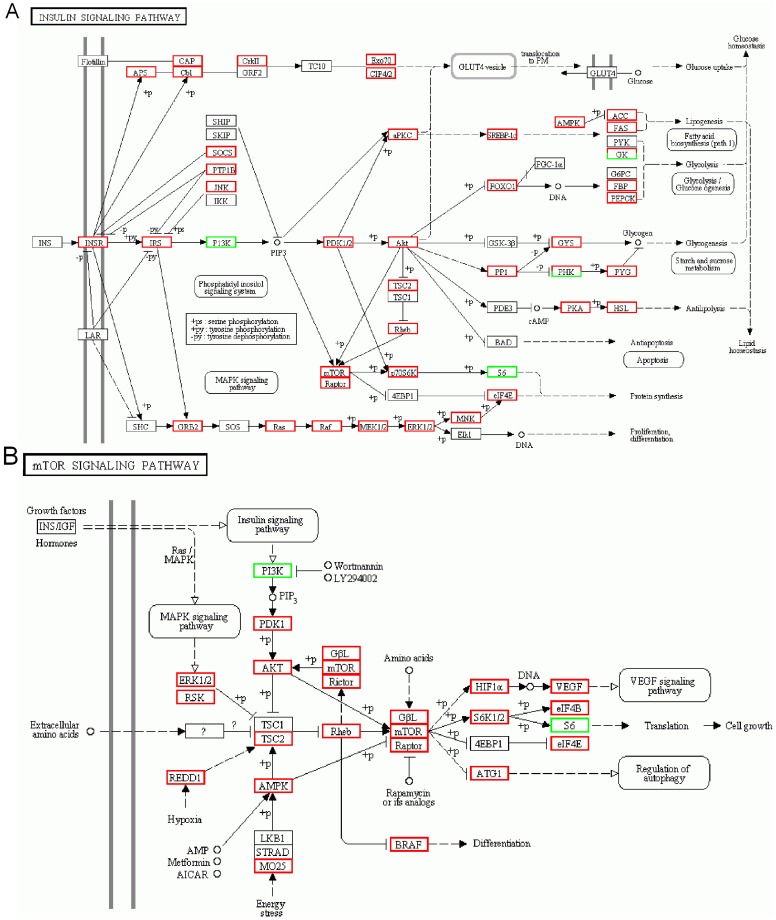
Insulin and mTOR signaling pathways affected by artificial diet feeding. (A) Insulin signaling pathway. (B) mTOR signaling pathway. Red and green frames indicate genes and enriched functions that were up- and down-regulated in artificial diet-fed (AD_1) and prey-fed (CY_1) treatments.

Insulin signaling plays a very important role in the regulation of glucose and lipid metabolism [67], and the insulin/TOR pathway is a conserved signaling cascade that functions as a nutrient sensing pathway by linking food-intake to animal growth and metabolism, including reproduction and lifespan [68], [69]. In honey bees, this pathway plays a major role in the regulation of aging of individuals [70]. Most genes in the insulin and mTOR signaling pathway were up-regulated in diet-fed insects (Figure 5), again indicating excess amounts of lipids and sugar in the diet. However, the PI 3-kinases (or PI3Ks) and the ribosomal protein S6 were down-regulated in diet-fed insects. Many PI3Ks have been linked to a diverse group of cellular functions, including cell growth, proliferation, differentiation, motility, survival and intracellular trafficking. Further, PI3Ks are a key component of the insulin signaling pathway [71], which regulates glucose uptake through a series of phosphorylation events.

The target-of-rapamycin (TOR) has been shown to respond to the presence of amino acids and induce up-regulation of ribosome biogenesis, translation [72]–[76] and energy metabolism [77], [78] required for tissue growth. Representing an extensively studied effector of the TOR complex 1 (TORC1), S6K possesses an important yet incompletely defined role in cellular and organismal physiology. TORC1 functions as an environmental sensor by integrating signals derived from diverse environmental cues to promote anabolic and inhibit catabolic cellular functions. Mammalian TORC1 (mTORC1) phosphorylates and activates S6K1 and S6K2, whose first identified substrate was ribosomal protein S6. The mTORC1-S6K1 axis controls fundamental cellular processes, including transcription, translation, protein and lipid synthesis, cell growth/size and metabolism, glucose homoeostasis, insulin sensitivity, adipocyte metabolism, body mass and energy balance, tissue and organ size [79]. For diet-fed *A. chinensis*, S6 in the mTOR signaling pathway was down-regulated (Figure 5), which may have caused delayed translation and cell growth, and probably contributed to the longer developmental time for nymphs.

In *D. melanogaster*, neurosecretory insulin-like peptide-producing cells (IPCs), analogous to mammalian pancreatic beta cells, are involved in glucose homeostasis. Extending those findings, Haselton *et al.* developed an oral glucose tolerance test in the adult fly and demonstrated that IPCs were responsible for executing an acute glucose clearance response. To further develop *D. melanogaster* as a relevant system for studying age-associated metabolic disorders, Haselton *et al.* determined the impact of adult-specific partial ablation of IPCs (IPC knockdown) on insulin-like peptide (ILP) action, metabolic outcomes and longevity. The findings showed a significant increase in stored glycogen and triglyceride levels as well as elevated levels of circulating lipids, increased resistance to starvation, impaired female fecundity, increased life span and decreased mortality; all of which demonstrated it was possible to modulate ILP action in adult flies to achieve life span extension without insulin resistance [80]. Based on this, we speculate that the lower fecundity and longer lifespan in diet-fed *A. chinensis* adults were probably linked to the high sucrose level in the artificial diet.

### Putative Molecular Markers

We predicted a total of 60,420 putative single nucleotide polymorphisms (SNPs) from CY_1 *A. chinensis* libraries wherein 22,759 were transversions and 37,661 were transitions; a total of 77,892 SNPs from AD_1 *A. chinensis* libraries wherein 27,776 were transversions and 50,116 were transitions (Table 1, Table S9, Table S10, Table S11, Table S12). In total, 4,115 simple sequence repeats (SSRs or microsatellites) including 697 (16.94%) dinucleotide, 592 (14.39%) trinucleotide repeats, and 34 (0.83%) pentanucleotide repeats were identified from All-unigenes of *A. chinensis*. Additionally, 3,814 All-unigenes contained SSRs, in which 269 (7.05%) had more than 1 SSR (Table 2, Table S13). Molecular markers identified in the current study could lay a platform for better understanding the adaptation/ecology of *A. chinensis*. However all the predicted molecular markers need to be validated to rule out false positives and sequencing errors.

**Table 1:** Predicted single nucleotide polymorphisms (SNP) in Chinese oak silk moth pupae-fed (CY_1) and artificial diet-fed (AD_1) *Arma chinensis* sequences.

SNP type	CY_1	AD_1
**Number of transition**	37,661	50,116
A–G	18,635	24,945
C–T	19,026	25,171
**Number of transversion**	22,759	27,776
A–C	5,216	6,290
A–T	8,654	10,507
C–G	3,551	4,586
G–T	5,338	6,393
**Total**	60,420	77,892

**Table 2:** Summary of microsatellite loci predicted in *Arma chinensis* sequences.

Number of repeats	Mononucleotide repeats	Dinucleotide repeats	Trinucleotide repeats	Tetranucleotide repeats	Pentanucleotide repeats	Hexanucleotide repeats
4	0	0	0	0	28	13
5	0	0	403	13	4	1
6	0	338	142	5	0	0
7	0	146	34	1	2	0
8	0	83	9	0	0	0
9	0	31	1	0	0	0
10	0	39	1	0	0	0
11	0	53	0	0	0	0
12	897	6	0	0	0	0
13	554	0	0	0	0	0
14	353	0	1	0	0	0
15	230	0	0	0	0	0
16	149	0	0	0	0	0
17	101	0	0	0	0	0
18	102	0	0	0	0	0
19	109	0	0	0	0	0
20	114	0	0	0	0	0
21	85	1	0	0	0	0
22	41	0	0	0	0	0
23	24	0	1	0	0	0
Subtotal	2,759	697	592	19	34	14

### Quantitative Real-time PCR (qRT-PCR) Validation

**Figure 6:**
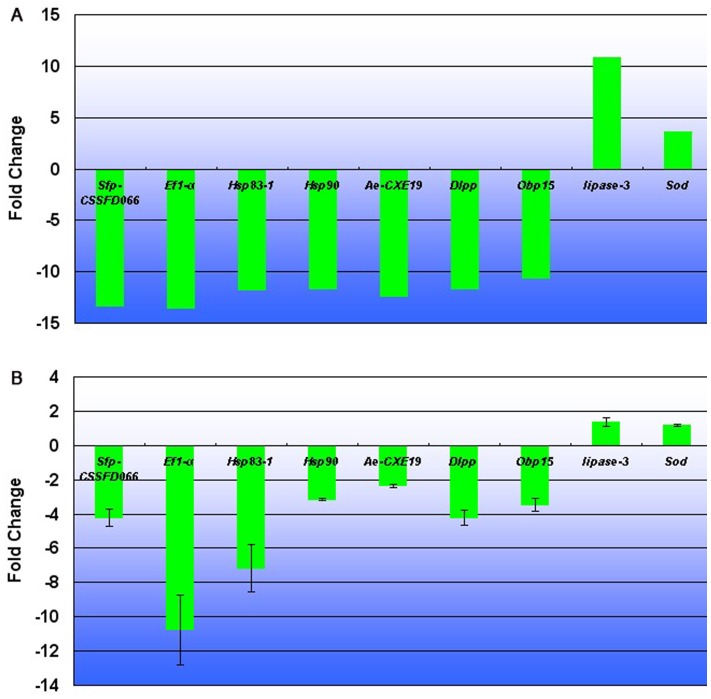
Verification of differentially expressed genes (DEG) by qRT-PCR. (A) DEG data in transcriptome analysis. The fold changes of the genes were calculated as the log2 value of each AD_1/CY_1 comparison and are shown on the y-axis. (B) The qRT-PCR analysis of gene expression data. Expression ratios of selected genes in AD-1 compared to CY_1.

To validate the differentially expressed genes (DEG) determined by the transcriptome results, we compared expression profiles of the CY_1 and AD_1 using qRT-PCR. We selected nine genes randomly, all of which demonstrated a concordant direction of change for both DEG and qRT-PCR. But both methods indicated only two genes, *elongation factor 1-alpha* (*Ef1-α*), *heat shock protein 83-1* (*Hsp83-1*), were down-regulated in AD_1 by ca. 10 times the amount found in CY_1. Other seven genes, *seminal fluid protein CSSFP066* (*Sfp-CSSFD066*), *heat shock protein 90* (*Hsp90*), *antennal esterase CXE19* (*Ae-CXE19*), *defensin-like protein precursor* (*Dlpp*), *odorant binding protein 15* (*Obp15*), *lipase-3*, and *Cu*, *Zn-superoxide dismutase* (*Sod*), were up- or down-regulated in AD_1 with lower values for the qRT-PCR method compared to transcriptome sequencing (Figure 6, Table S14). This difference might be caused by a lower sensitivity of qRT-PCR than transcriptome sequencing, and read coverage may be uneven across the transcript length, owing to sequencing biases. Nevertheless, qRT-PCR analysis confirmed the direction of change detected by transcriptome analysis, indicating that our results are reliable.

### Conclusions

In the present study, we sequenced and characterized the transcriptome in artificial diet-fed and Chinese oak silk moth pupae-fed *A. chinensis*. Among 13,872 DEGs with significantly differential expression levels (FDR ≤ 0.001 and |log_2_Ratio| ≥ 1), more genes (10,261) were up-regulated in diet-fed insects. Additionally, many metabolic pathways related to nutrition were up-regulated in diet-fed *A. chinensis,* in some cases indicating excess quantities of specific nutrients in the diet. With this study, we showed that a nutrigenomic approach holds promise for deciphering the impact of dietary changes in insects and for improving diet formulations. We showed that changes in gene expression caused by dietary changes were correlated to physiological differences observed in diet-fed and pupae-fed *A. chinensis*. Several differentially expressed genes related to these different physiological properties were found, such as heat shock protein 90 (reduced fecundity), seminal fluid protein (lower egg viability), juvenile hormone esterase (prolonged nymphal development time), Cu, Zn-superoxide dismutase (longer lifespan in adults), antennal esterase CXE19, and odorant binding protein 15 (higher cannibalism in adults). It is worth noting that transcriptome analyses enabled determination of effects on male performance (e.g., expression of seminal fluid proteins), which is not easily attained via life history analyses. Also of importance, we found some metabolic pathways related to nutrition and differentially expressed nutrient-controlled genes, from which a more informative feedback for diet formulation was obtained and the artificial diet could be more efficiently optimized. In addition, a number of SNPs and microsatellite markers were predicted, which upon validation could facilitate the identification of polymorphisms within *A. chinensis* populations.

## Materials and Methods

### Insects

**Figure 7:**
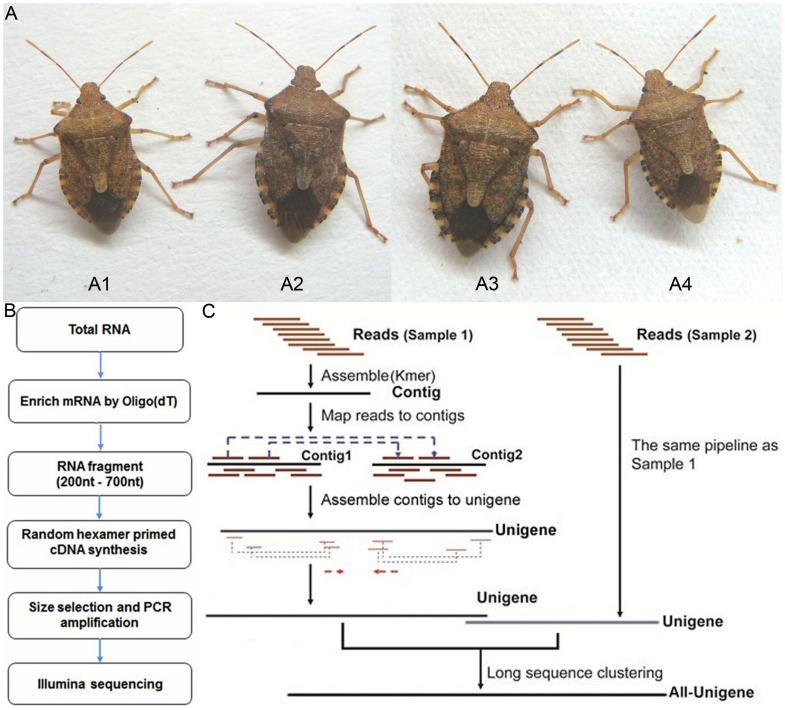
*Arma chinensis* fed with different diets and schematics of the transcriptome sequencing analysis. (A) Female adults fed with artificial diet (A1) and Chinese oak silk moth pupae (A2); male adults fed with artificial diet (A4) and Chinese oak silk moth pupae (A3). (B) Experiment pipeline of transcriptome. (C) Assembly process of the data.

The *A. chinensis* colonies were originally obtained from the Institute of Forest Protection, Jilin Provincial Academy of Forestry Sciences, Changchun, China. The insects used in this experiment (Figure 7A) were reared at 27±1°C and RH of 75±5%, and a 16∶8 (L:D) h photoperiod. The secondary prey, Chinese oak silk moth *A. pernyi* pupae, was purchased from a supermarket in Beijing and the artificial diet was comprised of pig liver, chicken egg and tuna but devoid of insect components as previously described. First to 5th instar nymphs were distinguished by appearance and body size. Adults used in this study were about 15 to 20 days old and were fertile, as verified by hatch of their eggs. A total of 200 eggs, and approximately 150 nymphs and adults were collected for RNA extraction. Total RNA was extracted from freshly sacrificed insects.

### cDNA Library Preparation and Illumina Sequencing for Transcriptome Analysis

Total RNA was extracted using RNAiso Plus (TaKaRa, Dalian, China) according to the manufacturer’s protocols. Gene expression information was obtained from RNA samples from eggs, 1st instar nymphs, 2nd instar nymphs, 3rd instar nymphs, 4th instar nymphs, 5th instar nymphs, and male and female adults obtained from the diet-fed and prey-fed treatments of the F_6_ and F_7_ generations, respectively.

The RNA sequencing was performed by the Beijing Genome Institute (Shenzhen, China), using a 2100 Bioanalyzer (Agilent Technologies) and following the Illumina manufacturer’s instructions. The poly(A)^+^ RNA was purified from 10 µg of pooled total RNA using oligo(dT) magnetic beads and fragmented into short sequences in the presence of divalent cations at 94°C for 5 min. The cleaved poly(A)^+^ RNA was transcribed, and then second-strand cDNA synthesis was performed. After the end-repair and ligation of adaptors, the products were amplified by PCR and purified using the QIA quick PCR purification kit to create a cDNA library, which was sequenced on the Illumina sequencing platform (HiSeq™ 2000). The sequencing machine generated raw images, which were controlled by the HCS system. The raw images were transformed into a BCL file by RTA, and BclConvert software was then used to generate raw reads from BCL (Figure 7B).

After removal of low quality reads, processed reads with an identity value of 95% and a coverage length of 90 bp were assembled using Trinity [46] *de novo* software and clustered using TGI clustering tools [81]. Trinity connected the contigs, forming unigenes sequences that could not be extended on either end. Because these two treatments were conducted with the same species, unigenes from each treatment assembly were processed further with sequence clustering software to acquire longer non-redundant All-unigenes (Figure 7C).

**Figure 8:**
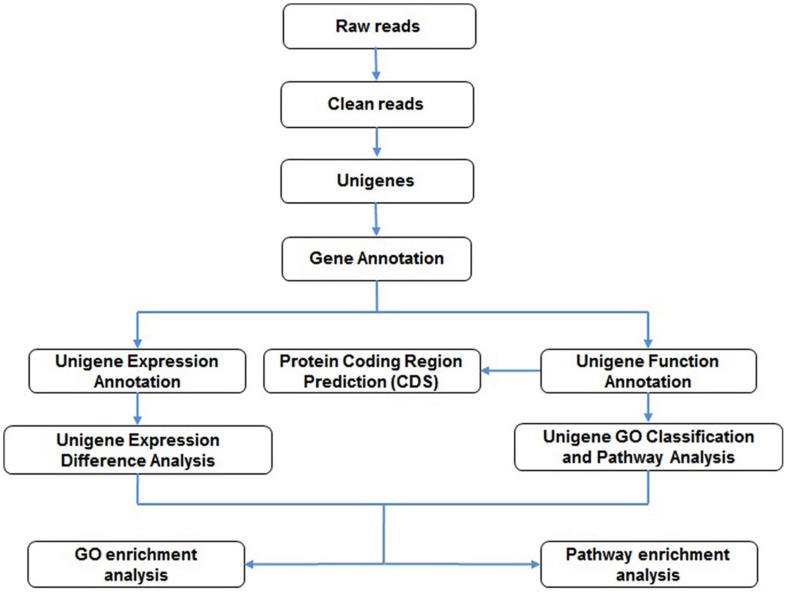
Pipeline of bioinformatics analysis.

For further analysis, we used BLASTx alignment (E-value <10^−5^) to search the unigene sequences against various protein databases such as Nr, Swiss-Prot, KEGG and COG. The BLAST results were used to extract CDS from the unigene sequences, and translate them into peptide sequences. The CDS of unigenes with no BLAST hits were predicted by ESTScan [52] and then translated into peptide sequences. For Nr annotation, we used the BLAST2GO program [82] and WEGO software [83] to explore the macro-distribution of gene functions for this species. A schematic of the pipeline used for the bioinformatics analysis is shown in Figure 8.

The FPKM method (fragments per kb per million fragments) [84] was used to calculate unigene expression. False discovery rate (FDR) was used to determine the threshold P-value in multiple tests. A FDR, 0.001 and an absolute value of the log2 ratio>1 were used as the threshold to determine significant differences in gene expression. The differentially expressed genes were used for GO and KO enrichment analyses. Enriched P values were calculated according to the hypergeometric test:
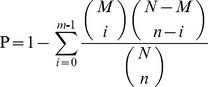
where N represents the number of genes with GO/KO annotation, n represents the number of differentially expressed genes in N, M represents the number of genes in each GO/KO term, and m represents the number of differentially expressed genes in each GO/KO term. For GO enrichment analysis, all P-values were performed with Bonferroni’s correction. We selected a corrected P-value <0.05 as the threshold to determine significant enrichment of the gene sets. For KO enrichment analysis, after multiple testing correction, we selected pathways with Q value ≤0.05, which were significantly enriched in DEGs. We considered the Q-values to be the FDR analogues of the P-values. The Q-value of an individual hypothesis test was the minimum FDR at which the test maybe called significant.

SSRs were identified with the Microsatellite identification tool (MISA) (http://pgrc.ipk-gatersleben.de/misa/). The script can identify both perfect and compound microsatellites, which are interrupted by a certain number of bases. SNPs were predicted using SOAPsnp software [85] with an arbitrary criterion of at least 2 reads supporting the consensus or variant.

### Quantitative Real-time PCR (qRT-PCR) Validation

Real-time PCR was performed on randomly selected genes expressed differentially (*heat shock protein 83-1, heat shock protein 90, seminal fluid protein CSSFP066, Cu, Zn-superoxide dismutase, antennal esterase CXE19, odorant binding protein 15, defensin-like protein precursor, Gl23315 (lipase 3)*, and *elongation factor 1-alpha*) with two biological replicates and three technical replications. Total RNA was extracted as described for the DEG library preparation and sequencing. The concentration of each RNA sample was adjusted to 1 µg/µl with nuclease-free water, and ca. 6 µg of total RNA was used as the template to synthesize first-strand cDNA in a 20 µl reaction system using a Superscript III Reverse Transcriptase kit (Invitrogen) following the manufacturer’s protocols. The sequences of the specific primer sets are listed in Table S15. The *bata actin* gene of *A. chinensis* was used as an internal gene. Quantitative real-time-PCR was performed using the SYBR(R) Green I Nucleic A kit (Invitrogen) according to the manufacturer’s protocols. The cycling parameters were 95°C for 2 min followed by 40 cycles at 95°C for 10 s and 60°C for 30 s ending with a melting curve analysis (60°C to 95°C in increments of 0.5°C every 5 s) to check for nonspecific product amplification. Relative gene expression was analyzed by the 2^−ΔΔCT^ method (Applied Biosystems 7500 Fast Real-Time PCR System).

## Supporting Information

Figure S1 **Overview of** ***Arma chinensis*** **transcriptome assembly (1).** (A) and (C) Size distribution of the contigs obtained from high-quality clean reads of AD_1 (*A. chinensis* fed on artificial diet ) and CY_1 (*A. chinensis* fed on Chinese oak silk moth pupae), respectively. (B) and (D) Size distribution of the unigenes produced from further assembly of contigs from AD_1 and CY_1, respectively.
(TIF)

Figure S2 **Overview of** ***Arma chinensis*** **transcriptome assembly (2).** (A) Size distribution of the All-unigenes produced from further assembly of AD_1 and CY_1 unigenes. (B) Size distribution of the CDS produced by searching All-unigene sequences against various protein databases (Nr, Swiss-Prot, KEGG and COG, in order) using BLASTx (E-value <10^−5^). (C) and (E) Size distributions of the ESTs and proteins obtained from the ESTScan results. (D) Size distribution of the proteins predicted from the CDS sequences.
(TIF)

Figure S3 **Characteristics of homology search of Illumina sequences against the nr database.** (A) E-value distribution of BLAST hits for each unique sequence with a cut-off E-value of 1.0E^−5^. (B) Similarity distribution of the top BLAST hits for each sequence. (C) Species distribution of unigenes top BLASTx results against the nr protein database with a cutoff E-value of at least 1.0E^−5^.
(TIF)

Table S1 Summary for the Chinese oak silk moth pupae-fed (CY_1) and artificial diet-fed (AD_1) *Arma chinensis* transcriptome.
(DOC)

Table S2 Gene Ontology of *Arma chinensis* sequences.
(XLS)

Table S3 KEGG summary of *Arma chinensis* sequences.
(XLS)

Table S4 Top thirty differentially expressed genes.
(DOC)

Table S5 GO of DEG in CY_1-vs-AD_1.
(XLS)

Table S6 Differentially expressed genes related to different biological characteristics.
(DOC)

Table S7 KEGG of DEG related to artificial diets.
(DOC)

Table S8 Differentially expressed nutrient-controlled genes (DENCG).
(DOC)

Table S9 Putative SNPs in CY_1 *Arma chinensis* (1).
(XLS)

Table S10 Putative SNPs in CY_1 *Arma chinensis* (2).
(XLS)

Table S11 Putative SNPs in AD_1 *Arma chinensis* (1).
(XLS)

Table S12 Putative SNPs in AD_1 *Arma chinensis* (2).
(XLS)

Table S13 Putative microsatellite loci in *Arma chinensis*.
(XLS)

Table S14 Verification of differentially expressed genes by qRT-PCR.
(XLS)

Table S15 Sequences of qRT-PCR primers.
(XLS)
