# Supplementary figures and images for: Nutrigenomics in Arma chinensis: Transcriptome Analysis of Arma chinensis Fed on Artificial Diet and Chinese Oak Silk Moth Antheraea pernyi Pupae
Source: PLoS One. 2013 Apr 11;8(4):e60881. doi: 10.1371/journal.pone.0060881 (PMC3623872; doi:10.1371/journal.pone.0060881)

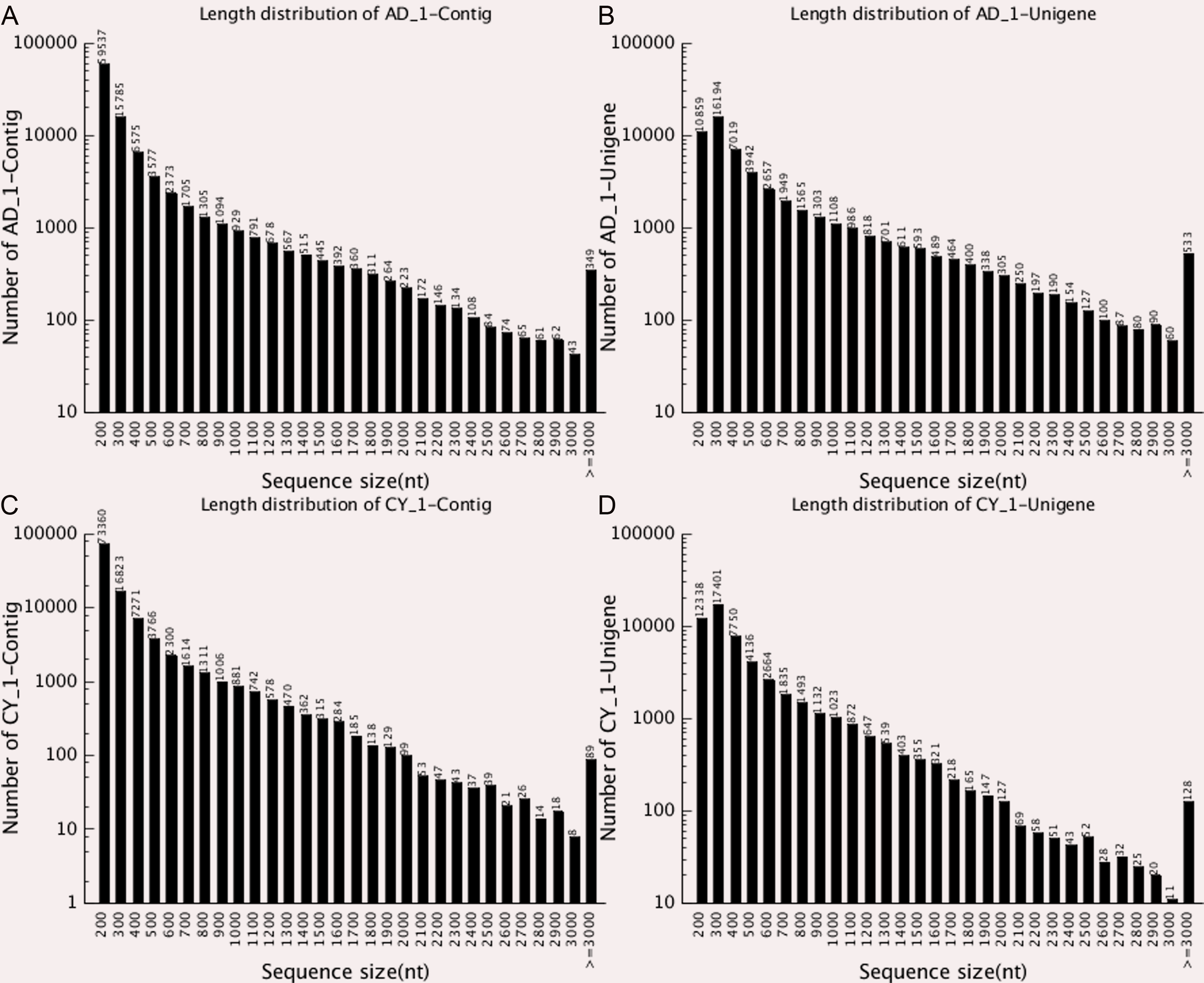

Supplement: Figure S1 — Overview of Arma chinensis transcriptome assembly (1). (A) and (C) Size distribution of the contigs obtained from high-quality clean reads of AD_1 (A. chinensis fed on artificial diet ) and CY_1 (A. chinensis fed on Chinese oak silk moth pupae), respectively. (B) and (D) Size distribution of the unigenes produced from further assembly of contigs from AD_1 and CY_1, respectively. (TIF) [file pone.0060881.s001.tif]

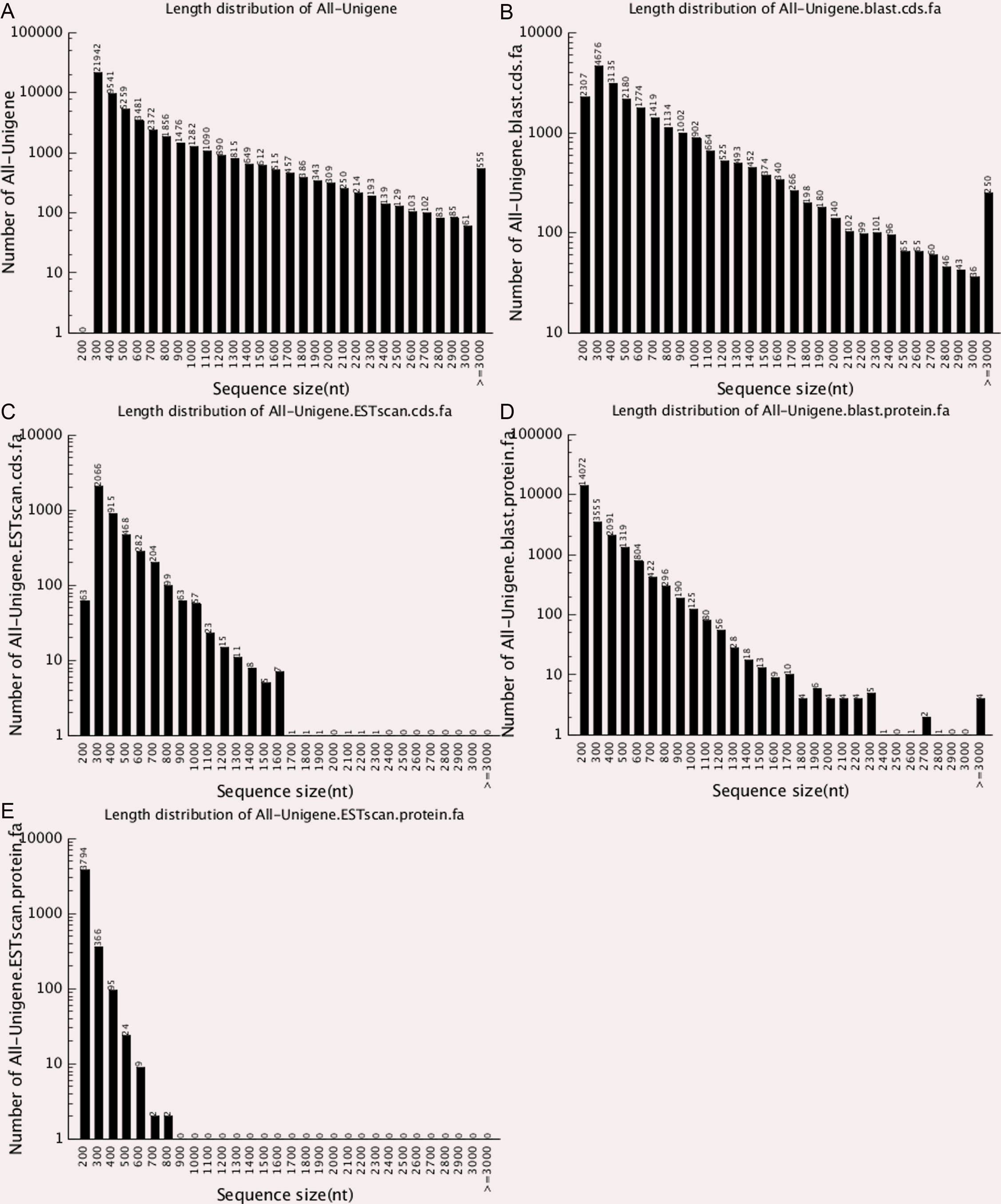

Supplement: Figure S2 — Overview of Arma chinensis transcriptome assembly (2). (A) Size distribution of the All-unigenes produced from further assembly of AD_1 and CY_1 unigenes. (B) Size distribution of the CDS produced by searching All-unigene sequences against various protein databases (Nr, Swiss-Prot, KEGG and COG, in order) using BLASTx (E-value <10−5). (C) and (E) Size distributions of the ESTs and proteins obtained from the ESTScan results. (D) Size distribution of the proteins predicted from the CDS sequences. (TIF) [file pone.0060881.s002.tif]

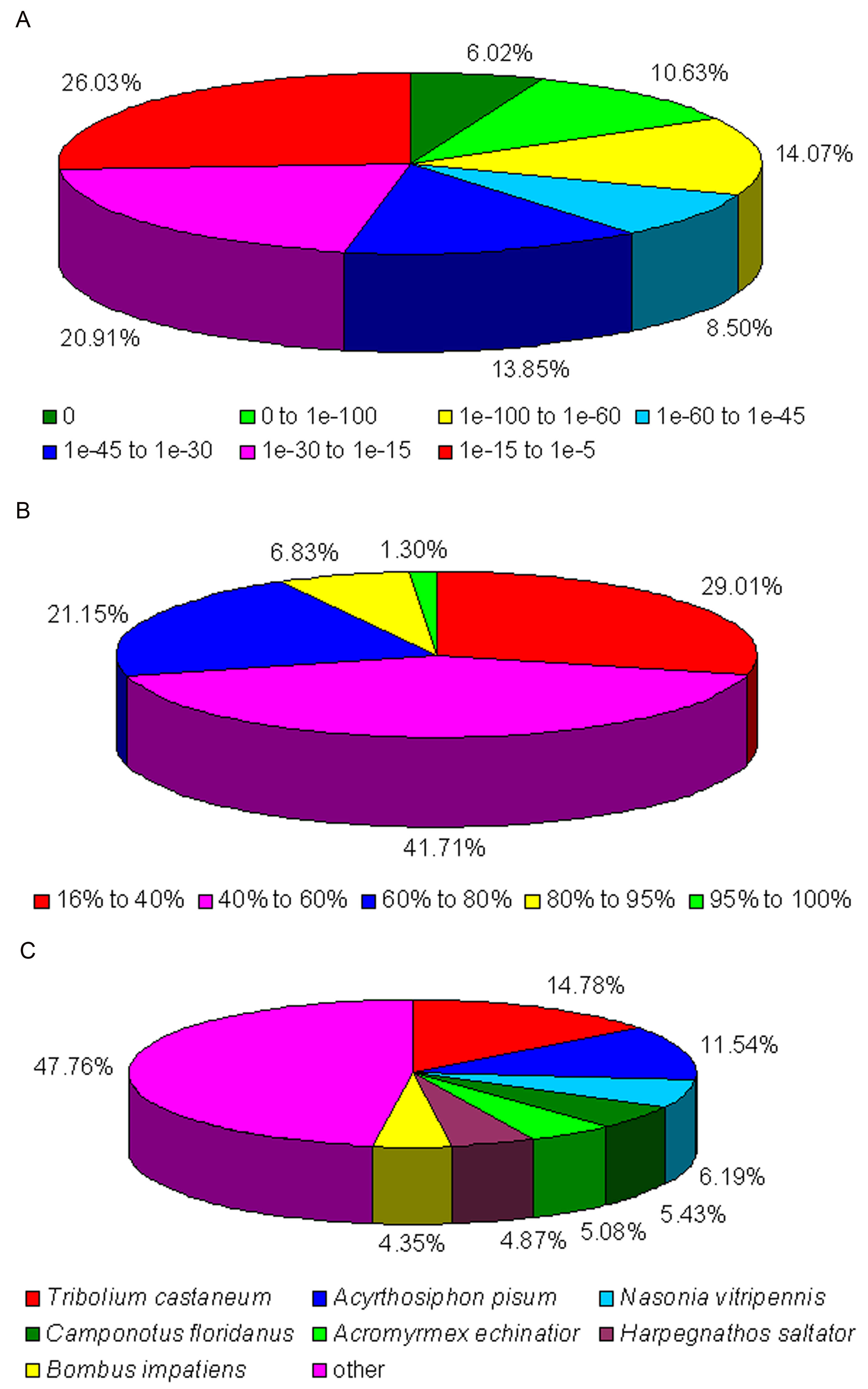

Supplement: Figure S3 — Characteristics of homology search of Illumina sequences against the nr database. (A) E-value distribution of BLAST hits for each unique sequence with a cut-off E-value of 1.0E−5. (B) Similarity distribution of the top BLAST hits for each sequence. (C) Species distribution of unigenes top BLASTx results against the nr protein database with a cutoff E-value of at least 1.0E−5. (TIF) [file pone.0060881.s003.tif]
