# Supplementary material for: Nutrigenomics in Arma chinensis: Transcriptome Analysis of Arma chinensis Fed on Artificial Diet and Chinese Oak Silk Moth Antheraea pernyi Pupae
Source: PLoS One. 2013 Apr 11;8(4):e60881. doi: 10.1371/journal.pone.0060881 (PMC3623872; doi:10.1371/journal.pone.0060881)
Supplement: Table S1 — Summary for the Chinese oak silk moth pupae-fed (CY_1) and artificial diet-fed (AD_1) Arma chinensis transcriptome. (DOC) [file pone.0060881.s004.doc]

**Table S1. Summary for the Chinese oak silk moth pupae-fed (CY_1) and artificial diet-fed (AD_1) *Arma chinensis* transcriptome.**

| **Summary** | **CY_1** | **AD_1** | **All*** |
| --- | --- | --- | --- |
| **Total Number of Raw Reads** | **63,639,102** | **62,031,248** | **-** |
| **Total Number of Clean Reads** | **53,224,704** | **52,244,538** | **-** |
| **Total Number of Clean Nucleotides (nt)**** | **4,790,223,360** | **4,702,008,420** | **-** |
| **Total Number of Contigs** | **112,029** | **98,724** | **-** |
| **Total Length (nt) of Contigs** | **28,024,077** | **30,459,937** | **-** |
| **Mean Length (nt) of Contigs** | **250** | **309** | **-** |
| **N50 of Contigs** | **318** | **494** | **-** |
| **Total Number of Unigenes** | **54,083** | **54,169** | **55,189** |
| **Total Length (nt) of Unigenes** | **23,080,277** | **29,326,931** | **32,153,970** |
| **Mean Length (nt) of Unigenes** | **427** | **541** | **583** |
| **N50 of Unigenes** | **541** | **869** | **818** |
| **Total Consensus Sequences** | **54,083** | **54,169** | **55,189** |
| **Distinct Clusters** | **8,636** | **10,315** | **12,046** |
| **Distinct Singletons** | **45,447** | **43,854** | **43,143** |

*** ‘All’ refers to the additional assembly of *A. chinensis* transcriptome from CY_1 and AD_1.**

****Total Clean Nucleotides = Total Clean Reads 1 × Read 1 Size + Total Clean Reads 2 × Read 2 Size.**
