# Supplementary material for: Nutrigenomics in Arma chinensis: Transcriptome Analysis of Arma chinensis Fed on Artificial Diet and Chinese Oak Silk Moth Antheraea pernyi Pupae
Source: PLoS One. 2013 Apr 11;8(4):e60881. doi: 10.1371/journal.pone.0060881 (PMC3623872; doi:10.1371/journal.pone.0060881)
Supplement: Table S4 — Top thirty differentially expressed genes. (DOC) [file pone.0060881.s007.doc]

**Table S4. Top thirty differentially expressed genes in libraries of *Arma chinensis* artificial diet-fed (AD_1) and Chinese oak silk moth pupae-fed (CY_1) treatments.**

| **Comparison** | **Unigene**  **ID** | **Hit Number** | **Discription** | **FDR** | **Fold Change** |
| --- | --- | --- | --- | --- | --- |
| **AD_1/CY_1**  **(up-regulated)** | **Unigene26203_All** | **gi|293340219|ref|XP_002724668.1|** | **PREDICTED: hypothetical protein [*Rattus norvegicus*] >gi|293351642|ref|XP_002727840.1| PREDICTED: hypothetical protein [*Rattus norvegicus*]** | **0** | **17.5568** |
|  | **Unigene26209_All** | **--** | **--** | **2.13E-262** | **16.5054** |
|  | **Unigene23689_All** | **gi\|157105546\|ref\|XP_001648917.1\|** | **hypothetical protein AaeL_AAEL014517 [*Aedes aegypti*] >gi\|108868998\|gb\|EAT33223.1\| conserved hypothetical protein [*Aedes aegypti*]** | **0** | **15.9997** |
|  | **Unigene32007_All** | **--** | **--** | **2.35E-78** | **15.6431** |
|  | **Unigene31994_All** | **gi\|326429708\|gb\|EGD75278.1\|** | **hypothetical protein PTSG_06930 [*Salpingoeca* *sp.* ATCC 50818]** | **2.16E-181** | **15.1392** |
|  | **Unigene23676_All** | **gi\|342316092\|gb\|ABF56562.2\|** | **cytochrome c oxidase subunit I [*Sus scrofa*]** | **1.05E-161** | **15.1281** |
|  | **Unigene32017_All** | **gi\|209166104\|gb\|ACI41483.1\|** | **cytochrome oxidase subunit II [*Sus scrofa*] >gi\|209166118\|gb\|ACI41496.1\| cytochrome oxidase subunit II [*Sus scrofa*] >gi\|209166132\|gb\|ACI41509.1\| cytochrome oxidase subunit II [*Sus scrofa*] >gi\|209166146\|gb\|ACI41522.1\| cytochrome oxidase subunit II [*Sus scrofa*] >gi\|209166160\|gb\|ACI41535.1\| cytochrome oxidase subunit II [*Sus scrofa*] >gi\|209166174\|gb\|ACI41548.1\| cytochrome oxidase subunit II [*Sus scrofa*] >gi\|209166188\|gb\|ACI41561.1\| cytochrome oxidase subunit II [*Sus scrofa*] >gi\|209166202\|gb\|ACI41574.1\| cytochrome oxidase subunit II [*Sus scrofa*] >gi\|209166230\|gb\|ACI41600.1\| cytochrome oxidase subunit II [*Sus scrofa*] >gi\|209166272\|gb\|ACI41639.1\| cytochrome oxidase subunit II [*Sus scrofa*]** | **3.81E-140** | **15.0606** |
|  | **Unigene32005_All** | **gi\|37719398\|gb\|AAR01785.1\|** | **ATP synthase F0 subunit 6 [*Sus scrofa*]** | **5.09E-142** | **14.6856** |
|  | **Unigene31649_All** | **--** | **--** | **2.87E-143** | **14.5966** |
|  | **Unigene26739_All** | **gi\|145315777\|gb\|ABP63312.1\|** | **NADH dehydrogenase subunit 1 [*Sus scrofa*]** | **1.47E-56** | **13.8048** |
|  | **Unigene26698_All** | **--** | **--** | **2.45E-32** | **13.7048** |
|  | **Unigene30583_All** | **gi\|157056528\|gb\|ABV02160.1\|** | **NADH dehydrogenase subunit 3 [*Sus scrofa*]** | **3.22E-25** | **13.671** |
|  | **CL4900.Contig1_All** | **gi\|145386514\|ref\|NP_001078814.1\|** | **apidermin 1 precursor [*Apis mellifera*]** | **2.25E-15** | **13.6344** |
|  | **Unigene30641_All** | **gi\|270002906\|gb\|EEZ99353.1\|** | **tollo [*Tribolium castaneum*]** | **3.30E-21** | **13.6229** |
|  | **Unigene30545_All** | **--** | **--** | **1.36E-20** | **13.4861** |
|  | **Unigene26722_All** | **gi\|312233373\|ref\|YP_004021584.1\|** | **NADH dehydrogenase subunit 4 [*Sus scrofa taiwanensis*] >gi\|83779141\|gb\|ABC47385.1\| NADH dehydrogenase subunit 4 [*Sus scrofa*] >gi\|125213072\|dbj\|BAF46412.1\| NADH dehydrogenase subunit 4 [*Sus scrofa*]**  **>gi\|145315438\|gb\|ABP62998.1\| NADH dehydrogenase subunit 4 [*Sus scrofa*] >gi\|145315452\|gb\|ABP63011.1\| NADH dehydrogenase subunit 4 [*Sus scrofa*] >gi\|145315466\|gb\|ABP63024.1\| NADH dehydrogenase subunit 4 [*Sus scrofa*]**  **>gi\|145315480\|gb\|ABP63037.1\| NADH dehydrogenase subunit 4 [*Sus scrofa*] >gi\|145315494\|gb\|ABP63050.1\| NADH dehydrogenase subunit 4 [*Sus scrofa*] >gi\|145315508\|gb\|ABP63063.1\|**  **NADH dehydrogenase subunit 4**  **[*Sus scrofa*] >gi\|145315522\|gb\|ABP63076.1\| NADH dehydrogenase subunit 4 [*Sus scrofa*] >gi\|145315536\|gb\|ABP63089.1\| NADH dehydrogenase subunit 4 [*Sus scrofa*] >gi\|145315550\|gb\|ABP63102.1\| NADH dehydrogenase subunit 4 [*Sus scrofa*] >gi\|145315564\|gb\|ABP63115.1\| NADH dehydrogenase subunit 4 [*Sus scrofa*] >gi\|145315578\|gb\|ABP63128.1\| NADH dehydrogenase subunit 4 [*Sus scrofa*] >gi\|145315592\|gb\|ABP63141.1\| NADH dehydrogenase subunit 4 [*Sus scrofa*] >gi\|145315606\|gb\|ABP63154.1\| NADH dehydrogenase subunit 4 [*Sus scrofa*] >gi\|145315620\|gb\|ABP63167.1\| NADH dehydrogenase subunit 4 [*Sus scrofa*] >gi\|145315634\|gb\|ABP63180.1\| NADH dehydrogenase subunit 4 [*Sus scrofa*] >gi\|145315648\|gb\|ABP63193.1\| NADH dehydrogenase subunit 4 [*Sus scrofa*] >gi\|145315662\|gb\|ABP63206.1\| NADH dehydrogenase subunit 4 [*Sus scrofa*] >gi\|145315676\|gb\|ABP63219.1\| NADH dehydrogenase subunit 4 [*Sus scrofa*] >gi\|145315690\|gb\|ABP63232.1\| NADH dehydrogenase subunit 4 [*Sus scrofa*] >gi\|145315704\|gb\|ABP63245.1\| NADH dehydrogenase subunit 4 [*Sus scrofa*] >gi\|145315718\|gb\|ABP63258.1\| NADH dehydrogenase subunit 4 [*Sus scrofa*] >gi\|145315732\|gb\|ABP63271.1\| NADH dehydrogenase subunit 4 [*Sus scrofa*] >gi\|145315746\|gb\|ABP63284.1\| NADH dehydrogenase subunit 4 [*Sus scrofa*] >gi\|145315760\|gb\|ABP63297.1\| NADH dehydrogenase subunit 4 [*Sus scrofa*] >gi\|145315800\|gb\|ABP63334.1\| NADH dehydrogenase subunit 4 [*Sus scrofa*] >gi\|157056545\|gb\|ABV02176.1\| NADH dehydrogenase subunit 4 [*Sus scrofa*] >gi\|225580662\|gb\|ABE02597.2\| NADH dehydrogenase subunit 4 [*Sus scrofa*] >gi\|238625878\|gb\|ACR48205.1\| NADH dehydrogenase subunit 4 [*Sus scrofa*] >gi\|269812094\|gb\|ACZ44406.1\| NADH dehydrogenase subunit 4 [*Sus scrofa taiwanensis*]** | **3.05E-104** | **13.3973** |
|  | **Unigene30622_All** | **--** | **--** | **3.14E-47** | **13.3574** |
|  | **Unigene30614_All** | **--** | **--** | **2.35E-78** | **13.2611** |
|  | **Unigene26700_All** | **gi\|33320783\|gb\|AAQ06072.1\|** | **NADH dehydrogenase subunit 2 [*Sus scrofa*] >gi\|33320825\|gb\|AAQ06111.1\| NADH dehydrogenase subunit 2 [*Sus scrofa*] >gi\|33320909\|gb\|AAQ06189.1\| NADH dehydrogenase subunit 2 [*Sus scrofa*] >gi\|80972994\|gb\|ABB53225.1\| NADH dehydrogenase subunit 2 [*Sus scrofa*]** | **3.89E-53** | **13.2453** |
|  | **Unigene24987_All** | **gi\|270014227\|gb\|EFA10675.1\|** | **kinesin heavy chain [*Tribolium castaneum*]** | **1.26E-12** | **13.1298** |
|  | **Unigene20936_All** | **--** | **--** | **8.37E-11** | **13.0846** |
|  | **Unigene32048_All** | **--** | **--** | **2.92E-42** | **13.0533** |
|  | **CL495.Contig2_All** | **--** | **--** | **9.43E-23** | **13.0349** |
|  | **Unigene33433_All** | **--** | **--** | **3.37E-10** | **12.9653** |
|  | **Unigene24956_All** | **gi\|345487497\|ref\|XP_001600183.2\|** | **PREDICTED: ras-related C3 botulinum toxin substrate 1 [*Nasonia vitripennis*]** | **9.21E-15** | **12.953** |
|  | **Unigene32996_All** | **--** | **--** | **4.16E-11** | **12.9422** |
|  | **Unigene22072_All** | **gi\|164614625\|gb\|ABY64538.1\|** | **alpha 1 S haptoglobin [*Sus scrofa*]** | **1.08E-27** | **12.9385** |
|  | **CL3120.Contig2_All** | **--** | **--** | **1.26E-12** | **12.9031** |
|  | **Unigene32229_All** | **gi\|328703354\|ref\|XP_001944392.2\|** | **PREDICTED: formin-like protein CG32138-like [*Acyrthosiphon pisum*]** | **4.71E-19** | **12.8991** |
|  | **Unigene22152_All** | **gi\|5835874\|ref\|NP_008645.1\|ND6_15069** | **NADH dehydrogenase subunit 6 [*Sus scrofa*] >gi\|223976090\|ref\|YP_002600788.1\| NADH dehydrogenase subunit 6 [*Sus scrofa domesticus*] >gi\|6093585\|sp\|O79882.1\|NU6M_PIG RecName: Full=NADH-ubiquinone oxidoreductase chain 6; AltName: Full=NADH dehydrogenase subunit 6 >gi\|4958963\|gb\|AAD34196.1\|AF034253_12 NADH dehydrogenase subunit 6 [*Sus scrofa*] >gi\|11055641\|gb\|AAG28188.1\|AF304200_12 NADH6 [*Sus scrofa*] >gi\|11055655\|gb\|AAG28201.1\|AF304201_12 NADH6 [*Sus scrofa*] >gi\|11055669\|gb\|AAG28214.1\|AF304202_12 NADH6 [*Sus scrofa*] >gi\|11055683\|gb\|AAG28227.1\|AF304203_12 NADH6 [*Sus scrofa*] >gi\|3702658\|emb\|CAA05238.1\| NADH dehydrogenase subunit 6 [*Sus scrofa*] >gi\|33320695\|gb\|AAQ05991.1\| NADH dehydrogenase subunit 6 [*Sus scrofa*] >gi\|33320709\|gb\|AAQ06004.1\| NADH dehydrogenase subunit 6 [*Sus scrofa*] >gi\|33320723\|gb\|AAQ06017.1\| NADH dehydrogenase subunit 6 [*Sus scrofa*] >gi\|33320737\|gb\|AAQ06030.1\| NADH dehydrogenase subunit 6 [*Sus scrofa*] >gi\|33320751\|gb\|AAQ06043.1\| NADH dehydrogenase subunit 6 [*Sus scrofa*] >gi\|33320765\|gb\|AAQ06056.1\| NADH dehydrogenase subunit 6 [*Sus scrofa*] >gi\|33320779\|gb\|AAQ06069.1\| NADH dehydrogenase subunit 6 [*Sus scrofa*] >gi\|33320793\|gb\|AAQ06082.1\| NADH dehydrogenase subunit 6 [*Sus scrofa*] >gi\|33320807\|gb\|AAQ06095.1\| NADH dehydrogenase subunit 6 [*Sus scrofa*] >gi\|33320821\|gb\|AAQ06108.1\| NADH dehydrogenase subunit 6 [*Sus scrofa*] >gi\|33320835\|gb\|AAQ06121.1\| NADH dehydrogenase subunit 6 [*Sus scrofa*] >gi\|33320849\|gb\|AAQ06134.1\| NADH dehydrogenase subunit 6 [*Sus scrofa*] >gi\|33320863\|gb\|AAQ06147.1\| NADH dehydrogenase subunit 6 [*Sus scrofa*] >gi\|33320877\|gb\|AAQ06160.1\| NADH dehydrogenase subunit 6 [*Sus scrofa*] >gi\|33320891\|gb\|AAQ06173.1\| NADH dehydrogenase subunit 6 [*Sus scrofa*] >gi\|33320905\|gb\|AAQ06186.1\| NADH dehydrogenase subunit 6 [*Sus scrofa*] >gi\|33320919\|gb\|AAQ06199.1\| NADH dehydrogenase subunit 6 [*Sus scrofa*] >gi\|33320933\|gb\|AAQ06212.1\| NADH dehydrogenase subunit 6 [*Sus scrofa*] >gi\|33320947\|gb\|AAQ06225.1\| NADH dehydrogenase subunit 6 [*Sus scrofa*] >gi\|33320961\|gb\|AAQ06238.1\| NADH dehydrogenase subunit 6 [*Sus scrofa*] >gi\|37719404\|gb\|AAR01791.1\| NADH dehydrogenase subunit 6 [*Sus scrofa*] >gi\|45826181\|gb\|AAS77702.1\| NADH dehydrogenase subunit 6 [*Sus scrofa*] >gi\|45826195\|gb\|AAS77715.1\| NADH dehydrogenase subunit 6 [*Sus scrofa*] >gi\|45826209\|gb\|AAS77728.1\| NADH dehydrogenase subunit 6 [*Sus scrofa*] >gi\|76262533\|gb\|ABA41421.1\| NADH dehydrogenase subunit 6 [*Sus scrofa*] >gi\|76262561\|gb\|ABA41447.1\| NADH dehydrogenase subunit 6 [*Sus scrofa*] >gi\|78499546\|gb\|ABB45799.1\| NADH dehydrogenase subunit 6 [*Sus scrofa*] >gi\|80973004\|gb\|ABB53235.1\| NADH dehydrogenase subunit 6 [*Sus scrofa*] >gi\|83779143\|gb\|ABC47387.1\| NADH dehydrogenase subunit 6 [*Sus scrofa*] >gi\|83779157\|gb\|ABC47400.1\| NADH dehydrogenase subunit 6 [*Sus scrofa*] >gi\|90969046\|gb\|ABE02599.1\| NADH dehydrogenase subunit 6 [*Sus scrofa*] >gi\|94981332\|gb\|ABF49560.1\| NADH dehydrogenase subunit 6 [*Sus scrofa*] >gi\|95116716\|gb\|ABF56571.1\| NADH dehydrogenase subunit 6 [*Sus scrofa*] >gi\|124249819\|gb\|ABM92892.1\| NADH dehydrogenase subunit 6 [*Sus scrofa*] >gi\|125213074\|dbj\|BAF46414.1\| NADH dehydrogenase subunit 6 [*Sus scrofa*] >gi\|145315440\|gb\|ABP63000.1\| NADH dehydrogenase subunit 6 [*Sus scrofa*] >gi\|145315454\|gb\|ABP63013.1\| NADH dehydrogenase subunit 6 [*Sus scrofa*] >gi\|145315468\|gb\|ABP63026.1\| NADH dehydrogenase subunit 6 [*Sus scrofa*] >gi\|145315482\|gb\|ABP63039.1\| NADH dehydrogenase subunit 6 [*Sus scrofa*] >gi\|145315496\|gb\|ABP63052.1\| NADH dehydrogenase subunit 6 [*Sus scrofa*] >gi\|145315510\|gb\|ABP63065.1\| NADH dehydrogenase subunit 6 [*Sus scrofa*] >gi\|145315524\|gb\|ABP63078.1\| NADH dehydrogenase subunit 6 [*Sus scrofa*] >gi\|145315538\|gb\|ABP63091.1\| NADH dehydrogenase subunit 6 [*Sus scrofa*] >gi\|145315552\|gb\|ABP63104.1\| NADH dehydrogenase subunit 6 [*Sus scrofa*] >gi\|145315566\|gb\|ABP63117.1\| NADH dehydrogenase subunit 6 [*Sus scrofa*] >gi\|145315580\|gb\|ABP63130.1\| NADH dehydrogenase subunit 6 [*Sus scrofa*] >gi\|145315608\|gb\|ABP63156.1\| NADH dehydrogenase subunit 6 [*Sus scrofa*] >gi\|145315622\|gb\|ABP63169.1\| NADH dehydrogenase subunit 6 [*Sus scrofa*] >gi\|145315636\|gb\|ABP63182.1\| NADH dehydrogenase subunit 6 [*Sus scrofa*] >gi\|145315650\|gb\|ABP63195.1\| NADH dehydrogenase subunit 6 [*Sus scrofa*] >gi\|145315692\|gb\|ABP63234.1\| NADH dehydrogenase subunit 6 [*Sus scrofa*] >gi\|145315720\|gb\|ABP63260.1\| NADH dehydrogenase subunit 6 [*Sus scrofa*] >gi\|145315734\|gb\|ABP63273.1\| NADH dehydrogenase subunit 6 [*Sus scrofa*] >gi\|145315748\|gb\|ABP63286.1\| NADH dehydrogenase subunit 6 [*Sus scrofa*] >gi\|145315762\|gb\|ABP63299.1\| NADH dehydrogenase subunit 6 [*Sus scrofa*] >gi\|145315802\|gb\|ABP63336.1\| NADH dehydrogenase subunit 6 [*Sus scrofa*] >gi\|156144892\|gb\|ABK62696.2\| NADH dehydrogenase subunit 6 [*Sus scrofa*] >gi\|157056437\|gb\|ABV02139.1\| NADH dehydrogenase subunit 6 [*Sus scrofa*] >gi\|157056532\|gb\|ABV02164.1\| NADH dehydrogenase subunit 6 [*Sus scrofa*] >gi\|157144257\|dbj\|BAF80082.1\| NADH dehydrogenase subunit 6 [*Sus scrofa*] >gi\|162423279\|gb\|ABX89420.1\| NADH dehydrogenase subunit 6 [*Sus scrofa*] >gi\|209166112\|gb\|ACI41491.1\| NADH dehydrogenase subunit 6 [*Sus scrofa*] >gi\|209166126\|gb\|ACI41504.1\| NADH dehydrogenase subunit 6 [*Sus scrofa*] >gi\|209166140\|gb\|ACI41517.1\| NADH dehydrogenase subunit 6 [*Sus scrofa*] >gi\|209166154\|gb\|ACI41530.1\| NADH dehydrogenase subunit 6 [*Sus scrofa*] >gi\|209166168\|gb\|ACI41543.1\| NADH dehydrogenase subunit 6 [*Sus scrofa*] >gi\|209166182\|gb\|ACI41556.1\| NADH dehydrogenase subunit 6 [*Sus scrofa*] >gi\|209166210\|gb\|ACI41582.1\| NADH dehydrogenase subunit 6 [*Sus scrofa*] >gi\|209166224\|gb\|ACI41595.1\| NADH dehydrogenase subunit 6 [*Sus scrofa*] >gi\|209166238\|gb\|ACI41608.1\| NADH dehydrogenase subunit 6 [*Sus scrofa*] >gi\|209166252\|gb\|ACI41621.1\| NADH dehydrogenase subunit 6 [*Sus scrofa*] >gi\|209166266\|gb\|ACI41634.1\| NADH dehydrogenase subunit 6 [*Sus scrofa*] >gi\|209166280\|gb\|ACI41647.1\| NADH dehydrogenase subunit 6 [*Sus scrofa*] >gi\|223972371\|dbj\|BAH23404.1\| NADH dehydrogenase subunit 6 [*Sus scrofa* *domestica*] >gi\|238625880\|gb\|ACR48207.1\| NADH dehydrogenase subunit 6 [*Sus scrofa*] >gi\|238625894\|gb\|ACR48220.1\| NADH dehydrogenase subunit 6 [*Sus scrofa*] >gi\|251829661\|gb\|ACT21214.1\| NADH dehydrogenase subunit 6 [*Sus scrofa*]** | **2.20E-27** | **12.7648** |
| **AD_1/CY_1**  **(down-regulated)** | **Unigene37512_All** | **--** | **--** | **0** | **-17.3529** |
|  | **Unigene42112_All** | **gi\|238694154\|ref\|YP_002922064.1\|** | **ATP synthase F0 subunit 6 [*Antheraea yamamai*] >gi\|189342962\|gb\|ACD91994.1\| ATP synthase F0 subunit 6 [*Antheraea yamamai*]** | **1.47E-55** | **-14.72** |
|  | **Unigene42154_All** | **--** | **--** | **9.92E-107** | **-14.3679** |
|  | **Unigene42126_All** | **gi\|238694161\|ref\|YP_002922071.1\|** | **cytochrome b [*Antheraea yamamai*] >gi\|189342969\|gb\|ACD92001.1\| cytochrome b [*Antheraea yamamai*]** | **3.16E-74** | **-14.2263** |
|  | **Unigene42135_All** | **--** | **--** | **3.11E-97** | **-14.1846** |
|  | **Unigene41833_All** | **--** | **--** | **1.16E-44** | **-14.1761** |
|  | **Unigene41784_All** | **gi\|340506702\|gb\|EGR32785.1\|** | **hypothetical protein IMG5_070670 [*Ichthyophthirius multifiliis*]** | **1.06E-102** | **-14.1626** |
|  | **Unigene38096_All** | **--** | **--** | **1.65E-25** | **-13.9938** |
|  | **Unigene38053_All** | **gi\|154343495\|ref\|XP_001567693.1\|** | **ribosomal protein l3 [*Leishmania braziliensis* MHOM/BR/75/M2904]** | **4.43E-26** | **-13.9167** |
|  | **Unigene40967_All** | **--** | **--** | **5.73E-19** | **-13.8012** |
|  | **Unigene38041_All** | **gi\|24415597\|gb\|AAN52157.1\|** | **cytochrome oxidase subunit II [*Antheraea pernyi*]** | **3.21E-27** | **-13.7521** |
|  | **Unigene38034_All** | **--** | **--** | **3.18E-25** | **-13.7218** |
|  | **CL3582.Contig2_All** | **gi\|90959527\|dbj\|BAE92616.1\|** | **PvLEA1 protein [*Polypedilum vanderplanki*]** | **8.66E-32** | **-13.6889** |
|  | **Unigene34025_All** | **gi\|71749502\|ref\|XP_828090.1\|** | **hypothetical protein [*Trypanosoma brucei* TREU927] >gi\|70833474\|gb\|EAN78978.1\| hypothetical protein Tb10.61.0020 [*Trypanosoma brucei brucei strain* 927/4 GUTat10.1]** | **4.48E-38** | **-13.6887** |
|  | **Unigene33997_All** | **gi\|322494579\|emb\|CBZ29881.1\|** | **beta tubulin [*Leishmania mexicana* MHOM/GT/2001/U1103]** | **1.80E-66** | **-13.6265** |
|  | **Unigene41798_All** | **gi\|154335074\|ref\|XP_001563777.1\|** | **elongation factor 1-alpha [*Leishmania braziliensis* MHOM/BR/75/M2904] >gi\|134060806\|emb\|CAM37822.1\| elongation factor 1-alpha [*Leishmania braziliensis* MHOM/BR/75/M2904]** | **3.58E-64** | **-13.5809** |
|  | **Unigene38027_All** | **--** | **--** | **8.61E-38** | **-13.5572** |
|  | **Unigene34782_All** | **gi\|71420711\|ref\|XP_811581.1\|** | **40S ribosomal protein S27 [*Trypanosoma cruzi strain* CL Brener] >gi\|71654414\|ref\|XP_815827.1\| 40S ribosomal protein S27 [*Trypanosoma cruzi strain* CL Brener] >gi\|71660305\|ref\|XP_821870.1\| ribosomal protein S27 [*Trypanosoma cruzi* strain CL Brener]** | **5.74E-19** | **-13.51** |
|  | **Unigene34820_All** | **--** | **--** | **8.23E-22** | **-13.4861** |
|  | **Unigene34832_All** | **gi\|3868784\|dbj\|BAA34219.1\|** | **MBF2 [*Samia cynthia*]** | **1.42E-15** | **-13.4305** |
|  | **Unigene38067_All** | **--** | **--** | **2.21E-98** | **-13.4084** |
|  | **Unigene38110_All** | **--** | **--** | **8.07E-50** | **-13.3917** |
|  | **Unigene38038_All** | **gi\|1764109\|gb\|AAC64661.1\|** | **pacifastin light chain precursor [*Pacifastacus leniusculus*]** | **1.66E-37** | **-13.3908** |
|  | **Unigene34831_All** | **--** | **--** | **7.81E-18** | **-13.3704** |
|  | **Unigene34301_All** | **--** | **--** | **7.42E-16** | **-13.3698** |
|  | **Unigene41003_All** | **gi\|364023679\|gb\|AEW46914.1\|** | **seminal fluid protein CSSFP066 [*Chilo suppressalis*]** | **2.99E-19** | **-13.3655** |
|  | **Unigene34212_All** | **gi\|154334610\|ref\|XP_001563552.1\|** | **60S ribosomal protein L6 [*Leishmania braziliensis* MHOM/BR/75/M2904]** | **2.18E-20** | **-13.2461** |
|  | **Unigene40938_All** | **--** | **--** | **4.07E-18** | **-13.2237** |
|  | **Unigene40997_All** | **gi\|357601811\|gb\|EHJ63159.1\|** | **serine protease inhibitor 28 [*Danaus plexippus*]** | **4.45E-28** | **-13.175** |
|  | **Unigene42685_All** | **--** | **--** | **8.51E-11** | **-13.1086** |

**The expression fold changes were performed with log 2 ratio.**
