# Supplementary material for: Nutrigenomics in Arma chinensis: Transcriptome Analysis of Arma chinensis Fed on Artificial Diet and Chinese Oak Silk Moth Antheraea pernyi Pupae
Source: PLoS One. 2013 Apr 11;8(4):e60881. doi: 10.1371/journal.pone.0060881 (PMC3623872; doi:10.1371/journal.pone.0060881)
Supplement: Table S6 — Differentially expressed genes related to different biological characteristics. (DOC) [file pone.0060881.s009.doc]

**Table S6. Differentially expressed genes related to different biological characteristics in libraries of *Arma chinensis* fed on artificial diet (AD_1) compared to Chinese oak silk moth pupae (CY_1).**

| **Biological**  **Characteristics in AD_1 compared to CY_1** | **Unigene**  **ID** | **Hit Number** | **Discription** | **FDR** | **Fold Change** |
| --- | --- | --- | --- | --- | --- |
| **reduced fecundity** | **Unigene41003_All^a^** | **gi|364023679|gb|AEW46914.1|** | **seminal fluid protein CSSFP066 [*Chilo suppressalis*]** | **2.99E-19** | **-13.3655** |
|  | **Unigene36113_All^a^** | **gi\|322494527\|emb\|CBZ29829.1\|** | **heat shock protein 83-1 [*Leishmania mexicana* MHOM/GT/2001/U1103] >gi\|322494528\|emb\|CBZ29830.1\| heat shock protein 83-1 [*Leishmania mexicana* MHOM/GT/2001/U1103] >gi\|322494529\|emb\|CBZ29831.1\| heat shock protein 83-1 [*Leishmania mexicana* MHOM/GT/2001/U1103]**  **Pathway: progesterone-mediated oocyte maturation** | **2.72E-05** | **-11.7664** |
|  | **Unigene39036_All^a^** | **gi\|291278246\|gb\|ADD91573.1\|** | **heat shock protein 90 [*Antheraea pernyi*]**  **Pathway: progesterone-mediated oocyte maturation** | **2.27E-06** | **-11.6524** |
|  | **Unigene38714_All^a^** | **gi\|322494527\|emb\|CBZ29829.1\|** | **heat shock protein 83-1 [*Leishmania mexicana* MHOM/GT/2001/U1103] >gi\|322494528\|emb\|CBZ29830.1\| heat shock protein 83-1 [*Leishmania mexicana* MHOM/GT/2001/U1103] >gi\|322494529\|emb\|CBZ29831.1\| heat shock protein 83-1 [*Leishmania mexicana* MHOM/GT/2001/U1103]**  **Pathway: progesterone-mediated oocyte maturation** | **9.70E-08** | **-11.4712** |
|  | **Unigene18963_All^b^** | **gi\|312190375\|dbj\|BAJ33507.1\|** | **vitellogenin [*Trigonotylus caelestialium*]** | **3.37E-25** | **2.0548** |
| **lower egg viability** | **Unigene41003_All^a^** | **gi|364023679|gb|AEW46914.1|** | **seminal fluid protein CSSFP066 [*Chilo suppressalis*]** | **2.99E-19** | **-13.3655** |
| **prolonged nymphal development time** | **Unigene27837_All^b^** | **gi|340712377|ref|XP_003394738.1|** | **PREDICTED: cytochrome P450 302a1, mitochondrial-like [*Bombus terrestris*]**  **ko: CYP302A1; ecdysteroid 22-hydroxylase** | **3.37E-09** | **3.5071** |
|  | **CL2154.Contig1_All^b^** | **gi\|328705775\|ref\|XP_001947452.2\|** | **PREDICTED: esterase FE4-like [*Acyrthosiphon pisum*]**  **ko: juvenile-hormone esterase [EC: 3.1.1.59]** | **7.38E-30** | **3.4027** |
|  | **CL2169.Contig1_All^b^** | **gi\|193610695\|ref\|XP_001951107.1\|** | **PREDICTED: esterase FE4-like isoform 1 [*Acyrthosiphon pisum*] >gi\|328721620\|ref\|XP_003247358.1\| PREDICTED: esterase FE4-like isoform 2 [*Acyrthosiphon pisum*] >gi\|328721622\|ref\|XP_003247359.1\| PREDICTED: esterase FE4-like isoform 3 [*Acyrthosiphon pisum*]**  **ko: juvenile-hormone esterase [EC: 3.1.1.59]** | **9.30E-13** | **3.1469** |
|  | **Unigene9442_All^b^** | **gi\|195963357\|ref\|NP_001124351.1\|** | **beta-esterase 2 precursor [*Bombyx mori*] >gi\|189916561\|gb\|ACE62800.1\| carboxylesterase CarE-15 [*Bombyx mori*]**  **ko: juvenile-hormone esterase [EC: 3.1.1.59]** | **3.05E-12** | **3.1031** |
|  | **Unigene32095_All^b^** | **gi\|328721613\|ref\|XP_001950655.2\|** | **PREDICTED: esterase FE4-like [*Acyrthosiphon pisum*]**  **ko: juvenile-hormone esterase [EC: 3.1.1.59]** | **1.48E-09** | **2.9739** |
|  | **CL2154.Contig2_All^b^** | **gi\|193579936\|ref\|XP_001950765.1\|** | **PREDICTED: venom carboxylesterase-6-like [*Acyrthosiphon pisum*]**  **ko: juvenile-hormone esterase [EC: 3.1.1.59]** | **2.44E-35** | **2.8813** |
|  | **CL4583.Contig1_All^b^** | **gi\|193579936\|ref\|XP_001950765.1\|** | **PREDICTED: venom carboxylesterase-6-like [*Acyrthosiphon pisum*]**  **ko: juvenile-hormone esterase [EC: 3.1.1.59]** | **7.11E-34** | **2.6725** |
|  | **Unigene2365_All^b^** | **gi\|62002225\|gb\|AAX58712.1\|** | **pheromone-degrading enzyme 2 [*Antheraea polyphemus*]**  **ko: juvenile-hormone esterase [EC: 3.1.1.59]** | **1.44E-26** | **2.2867** |
|  | **Unigene20273_All^b^** | **gi\|193579936\|ref\|XP_001950765.1\|** | **PREDICTED: venom carboxylesterase-6-like [*Acyrthosiphon pisum*]**  **ko: juvenile-hormone esterase [EC: 3.1.1.59]** | **6.27E-42** | **2.2106** |
|  | **CL1448.Contig1_All^b^** | **gi\|193610695\|ref\|XP_001951107.1\|** | **PREDICTED: esterase FE4-like isoform 1 [*Acyrthosiphon pisum*] >gi\|328721620\|ref\|XP_003247358.1\| PREDICTED: esterase FE4-like isoform 2 [*Acyrthosiphon pisum*] >gi\|328721622\|ref\|XP_003247359.1\| PREDICTED: esterase FE4-like isoform 3 [*Acyrthosiphon pisum*]**  **ko: juvenile-hormone esterase [EC: 3.1.1.59]** | **1.77E-06** | **2.0013** |
| **longer lifespan in adults** | **CL1970.Contig2_All^b^** | **gi|242247393|ref|NP_001156153.1|** | **superoxide dismutase [Cu-Zn]-like precursor [*Acyrthosiphon pisum*] >gi|239789311|dbj|BAH71286.1| ACYPI003921 [*Acyrthosiphon pisum*]**  **Swissprot: superoxide dismutase [Cu-Zn] OS = *Halocynthia roretzi* PE = 1 SV = 2** | **1.74E-12** | **3.6169** |
|  | **CL1970.Contig1_All^b^** | **gi\|307930990\|dbj\|BAJ21357.1\|** | **Cu, Zn-superoxide dismutase [*Polyandrocarpa misakiensis*]** | **6.31E-95** | **2.3664** |
|  | **CL3968.Contig2_All^b^** | **gi\|432542\|gb\|AAB28239.1\|** | **sodium pump alpha subunit [*Ctenocephalides felis*]**  **Swissprot: sodium/potassium-transporting ATPase subunit alpha OS = *Drosophila melanogaster* GN = Atpalpha PE = 1 SV = 3** | **8.94E-16** | **2.2159** |
| **higher cannibalism in adults** | **Unigene36533_All^a^** | **gi|357624776|gb|EHJ75423.1|** | **antennal esterase CXE19 [*Danaus plexippus*]** | **2.35E-11** | **-12.3625** |
|  | **Unigene38400_All^a^** | **gi\|6560637\|gb\|AAF16696.1\|AF117574_1** | **sensory appendage protein 1 [*Manduca sexta*]** | **4.24E-06** | **-11.7172** |
|  | **Unigene35579_All^a^** | **gi\|112983704\|ref\|NP_001037370.1\|** | **defensin-like protein precursor [*Bombyx mori*] >gi\|71564291\|gb\|AAZ38358.1\| defensin-like protein [*Bombyx mori*]** | **2.72E-05** | **-11.6452** |
|  | **Unigene40410_All^a^** | **gi\|270015618\|gb\|EFA12066.1\|** | **odorant binding protein 15 [*Tribolium castaneum*]** | **5.78E-04** | **-10.6318** |

**The corner marks of gene ID: ‘a’ represents down-regulated genes; ‘b’ represents up-regulated genes. The expression fold changes were performed with log 2 ratio and |log_2_Ratio| ≥ 2.**
