# Supplementary material for: Nutrigenomics in Arma chinensis: Transcriptome Analysis of Arma chinensis Fed on Artificial Diet and Chinese Oak Silk Moth Antheraea pernyi Pupae
Source: PLoS One. 2013 Apr 11;8(4):e60881. doi: 10.1371/journal.pone.0060881 (PMC3623872; doi:10.1371/journal.pone.0060881)
Supplement: Table S7 — KEGG of DEG related to artificial diets. (DOC) [file pone.0060881.s010.doc]

**Table S7. KEGG of DEG related to nutrients of artificial diets.**

| **Nutrients** | **Pathway** | **DEGs genes with pathway annotation (5879)** | **All genes with pathway annotation (15416)** | **P value** | **Q value** | **Pathway ID** |
| --- | --- | --- | --- | --- | --- | --- |
| **amino acid** | **alanine, aspartate and glutamate metabolism** | **38 (0.65%) (38↑, 0↓)** | **65 (0.42%)** | **0.0006904038** | **0.035492222** | **ko00250** |
|  | **glycine, serine and threonine metabolism** | **51 (0.87%) (50↑, 1↓)** | **99 (0.64%)** | **0.004505306** | **0.107676813** | **ko00260** |
|  | **arginine and proline metabolism** | **54 (0.92%) (51↑, 3↓)** | **114 (0.74%)** | **0.0271844** | **0.270711317** | **ko00330** |
|  | **valine, leucine and isoleucine degradation** | **40 (0.68%) (38↑, 2↓)** | **86 (0.56%)** | **0.06893573** | **0.365755592** | **ko00280** |
|  | **beta-alanine metabolism** | **26 (0.44%) (24↑, 2↓)** | **56 (0.36%)** | **0.1272194** | **0.490551355** | **ko00410** |
|  | **phenylalanine metabolism** | **20 (0.34%) (20↑, 0↓)** | **43 (0.28%)** | **0.1645595** | **0.564069363** | **ko00360** |
|  | **valine, leucine and isoleucine biosynthesis** | **17 (0.29%) (17↑, 0↓)** | **38 (0.25%)** | **0.2488403** | **0.615074877** | **ko00290** |
|  | **phenylalanine, tyrosine and tryptophan biosynthesis** | **3 (0.05%) (3↑, 0↓)** | **7 (0.05%)** | **0.5389458** | **0.934218441** | **ko00400** |
|  | **D-arginine and D-ornithine metabolism** | **3 (0.05%) (3↑, 0↓)** | **8 (0.05%)** | **0.6445125** | **0.999999700** | **ko00472** |
|  | **tryptophan metabolism** | **30 (0.51%) (30↑, 0↓)** | **88 (0.57%)** | **0.8136908** | **0.999999700** | **ko00380** |
|  | **histidine metabolism** | **11 (0.19%) (11↑, 0↓)** | **37 (0.24%)** | **0.8910307** | **0.999999700** | **ko00340** |
|  | **tyrosine metabolism** | **38 (0.65%) (37↑, 1↓)** | **120 (0.78%)** | **0.9419805** | **0.999999700** | **ko00350** |
|  | **cysteine and methionine metabolism** | **20 (0.34%) (19↑, 1↓)** | **70 (0.45%)** | **0.9641992** | **0.999999700** | **ko00270** |
|  | **lysine degradation** | **78 (1.33%) (69↑, 9↓)** | **267 (1.73%)** | **0.9991658** | **0.999999700** | **ko00310** |
| **fat** | **adipocytokine signaling pathway** | **49 (0.83%) (49↑, 0↓)** | **98 (0.64%)** | **0.01085965** | **0.144192019** | **ko04920** |
|  | **pyruvate metabolism** | **48 (0.82%) (47↑, 1↓)** | **103 (0.67%)** | **0.04828879** | **0.332813873** | **ko00620** |
|  | **fatty acid biosynthesis** | **21 (0.36%) (21↑, 0↓)** | **48 (0.31%)** | **0.2548220** | **0.621453653** | **ko00061** |
|  | **glycerolipid metabolism** | **61 (1.04%) (58↑,3↓)** | **169 (1.1%)** | **0.7340082** | **0.999999700** | **ko00561** |
|  | **fat digestion and absorption** | **48 (0.82%) (46↑, 2↓)** | **117 (0.76%)** | **0.2893001** | **0.668376930** | **ko04975** |
|  | **fatty acid metabolism** | **28 (0.48%) (27↑, 1↓)** | **67 (0.43%)** | **0.3091071** | **0.671605426** | **ko00071** |
|  | **fatty acid elongation** | **9 (0.15%) (8↑, 1↓)** | **21 (0.14%)** | **0.4062243** | **0.809063397** | **ko00062** |
| **starch and sugar** | **starch and sucrose metabolism** | **83 (1.41%) (79↑, 4↓)** | **201 (1.3%)** | **0.1959547** | **0.578187325** | **ko00500** |
|  | **carbohydrate digestion and absorption** | **49 (0.83%) (43↑, 6↓)** | **136 (0.88%)** | **0.7230465** | **0.999999700** | **ko04973** |
|  | **fructose and mannose metabolism** | **31 (0.53%) (29↑, 2↓)** | **95 (0.62%)** | **0.8884854** | **0.999999700** | **ko00051** |
| **vitamin** | **ascorbate and aldarate metabolism** | **37 (0.63%) (36↑, 1↓)** | **75 (0.49%)** | **0.0311286** | **0.286143669** | **ko00053** |
|  | **vitamin digestion and absorption** | **58 (0.99%) (55↑, 3↓)** | **135 (0.88%)** | **0.1423076** | **0.515326006** | **ko04977** |
|  | **folate biosynthesis** | **22 (0.37%) (20↑, 2↓)** | **50 (0.32%)** | **0.237461** | **0.603757223** | **ko00790** |
|  | **pantothenate and CoA biosynthesis** | **13 (0.22%) (13↑, 0↓)** | **29 (0.19%)** | **0.2874582** | **0.668376930** | **ko00770** |
|  | **nicotinate and nicotinamide metabolism** | **11 (0.19%) (10↑, 1↓)** | **37 (0.24%)** | **0.8910307** | **0.999999700** | **ko00760** |
|  | **biotin metabolism** | **4 (0.07%) (4↑, 0↓)** | **5 (0.03%)** | **0.07345719** | **0.365755592** | **ko00780** |
|  | **retinol metabolism** | **47 (0.8%) (46↑, 1↓)** | **106 (0.69%)** | **0.1119813** | **0.453619164** | **ko00830** |
|  | **thiamine metabolism** | **3 (0.05%) (3↑, 0↓)** | **7 (0.05%)** | **0.5389458** | **0.934218441** | **ko00730** |
|  | **vitamin B6 metabolism** | **5 (0.09%) (5↑, 0↓)** | **15 (0.1%)** | **0.7368429** | **0.999999700** | **ko00750** |
|  | **riboflavin metabolism** | **10 (0.17%) (10↑, 0↓)** | **30 (0.19%)** | **0.7648585** | **0.999999700** | **ko00740** |

**↑ and ↓ indicate genes that are up- or down-regulated.**
