# Supplementary material for: Nutrigenomics in Arma chinensis: Transcriptome Analysis of Arma chinensis Fed on Artificial Diet and Chinese Oak Silk Moth Antheraea pernyi Pupae
Source: PLoS One. 2013 Apr 11;8(4):e60881. doi: 10.1371/journal.pone.0060881 (PMC3623872; doi:10.1371/journal.pone.0060881)
Supplement: Table S8 — Differentially expressed nutrient-controlled genes (DENCG). (DOC) [file pone.0060881.s011.doc]

**Table S8. Differentially expressed nutrient-controlled genes (DENCG) in library of *Arma chinensis* fed on artificial diet (AD_1) compared to Chinese oak silk moth pupae (CY_1).**

| **DENCG** | **Unigene**  **ID** | **Hit Number** | **Discription** | **FDR** | **Fold Change** |
| --- | --- | --- | --- | --- | --- |
| **sugar-lipase-3** | **CL230.Contig1_All^a^** | **gi|195117518|ref|XP_002003294.1|** | **GI23315 [*Drosophila mojavensis*] >gi|193913869|gb|EDW12736.1| GI23315 [*Drosophila mojavensis*]**  **Swissprot: lipase 3 OS = *Drosophila melanogaster* GN = Lip3 PE = 2 SV = 1** | **1.02E-05** | **10.85** |
|  | **Unigene24961_All^a^** | **gi\|195386698\|ref\|XP_002052041.1\|** | **GJ23823 [*Drosophila virilis*] >gi\|194148498\|gb\|EDW64196.1\| GJ23823 [*Drosophila virilis*]**  **Swissprot: lipase 3 OS = *Drosophila melanogaster* GN = Lip3 PE = 2 SV = 1** | **1.23E-11** | **4.1106** |
|  | **CL5196.Contig2_All^a^** | **gi\|307203867\|gb\|EFN82798.1\|** | **lipase 3 [*Harpegnathos saltator*]** | **7.85E-05** | **3.4729** |
|  | **Unigene30550_All^a^** | **gi\|345481631\|ref\|XP_001605683.2\|** | **PREDICTED: lipase 3-like [*Nasonia vitripennis*]** | **1.56E-17** | **2.995** |
|  | **Unigene5922_All^a^** | **gi\|340721119\|ref\|XP_003398973.1\|** | **PREDICTED: lipase 3-like [*Bombus terrestris*]** | **9.02E-12** | **2.4777** |
|  | **Unigene10507_All^a^** | **gi\|328719670\|ref\|XP_001952539.2\|** | **PREDICTED: lipase 3-like [*Acyrthosiphon pisum*]** | **2.11E-72** | **2.2583** |
|  | **CL3090.Contig1_All^a^** | **gi\|307213910\|gb\|EFN89157.1\|** | **Lipase 3 [*Harpegnathos saltator*]** | **2.47E-12** | **2.0149** |
| **glucose transporter** | **Unigene26824_All^a^** | **gi|328699079|ref|XP_001946696.2|** | **PREDICTED: facilitated trehalose transporter Tret1-like [*Acyrthosiphon pisum*]**  **ko: MFS transporter, SP family, solute carrier family 2 (facilitated glucose transporter), member 8** | **9.20E-05** | **4.2507** |
|  | **Unigene25825_All^a^** | **gi\|195401370\|ref\|XP_002059286.1\|** | **GJ18154 [*Drosophila virilis*] >gi\|194142292\|gb\|EDW58698.1\| GJ18154 [*Drosophila virilis*]**  **ko: MFS transporter, SP family, solute carrier family 2 (facilitated glucose transporter), member 8** | **4.44E-11** | **4.0505** |
|  | **Unigene26732_All^a^** | **gi\|328713890\|ref\|XP_001946479.2\|** | **PREDICTED: facilitated trehalose transporter Tret1-like [*Acyrthosiphon pisum*]**  **ko: MFS transporter, SP family, solute carrier family 2 (facilitated glucose transporter), member 8** | **2.46E-19** | **3.0946** |
|  | **Unigene27878_All^a^** | **gi\|328701837\|ref\|XP_001944504.2\|** | **PREDICTED: facilitated trehalose transporter Tret1-like [*Acyrthosiphon pisum*]**  **ko: MFS transporter, SP family, solute carrier family 2 (facilitated glucose transporter), member 8** | **1.65E-06** | **2.8882** |
|  | **Unigene19366_All^a^** | **gi\|328699079\|ref\|XP_001946696.2\|** | **PREDICTED: facilitated trehalose transporter Tret1-like [*Acyrthosiphon pisum*]**  **ko: MFS transporter, SP family, solute carrier family 2 (facilitated glucose transporter), member 8** | **5.35E-22** | **2.8552** |
|  | **Unigene28227_All^a^** | **gi\|350409155\|ref\|XP_003488630.1\|** | **PREDICTED: facilitated trehalose transporter Tret1-like [*Bombus impatiens*]**  **ko: MFS transporter, SP family, solute carrier family 2 (facilitated glucose transporter), member 8** | **5.06E-06** | **2.8033** |
|  | **Unigene23592_All^a^** | **gi\|189241434\|ref\|XP_972450.2\|** | **PREDICTED: similar to glucose transporter (sugar transporter [*Tribolium castaneum*]**  **ko: MFS transporter, SP family, solute carrier family 2 (facilitated glucose transporter), member 1** | **2.09E-06** | **2.5589** |
|  | **CL4433.Contig1_All^a^** | **gi\|291461577\|dbj\|BAI83423.1\|** | **sugar transporter 9 [*Nilaparvata lugens*]**  **ko: facilitated trehalose transporter** | **2.59E-83** | **2.4293** |
|  | **Unigene7545_All^a^** | **gi\|170054022\|ref\|XP_001862939.1\|** | **sugar transporter [*Culex quinquefasciatus*] >gi\|167874409\|gb\|EDS37792.1\| sugar transporter [*Culex quinquefasciatus*]**  **ko: MFS transporter, SP family, solute carrier family 2 (facilitated glucose transporter), member 8** | **3.65E-10** | **2.3439** |
|  | **CL3498.Contig3_All^a^** | **gi\|340716118\|ref\|XP_003396549.1\|** | **PREDICTED: glucose transporter type 1-like [*Bombus terrestris*]**  **ko: MFS transporter, SP family, solute carrier family 2 (facilitated glucose transporter), member 1** | **3.10E-18** | **2.3102** |
| **insulin receptor** | **Unigene26998_All^a^** | **gi|307179528|gb|EFN67842.1|** | **Insulin receptor substrate 1-B [*Camponotus floridanus*]**  **ko: insulin receptor substrate** | **2.63E-05** | **4.4032** |
|  | **Unigene33149_All^a^** | **gi\|270015135\|gb\|EFA11583.1\|** | **insulin-like receptor [*Tribolium castaneum*]**  **ko: insulin receptor [EC:2.7.10.1]** | **5.59E-11** | **2.6874** |
| **fatty acid**  **synthase** | **Unigene27521_All^a^** | **gi|328721179|ref|XP_001949072.2|** | **PREDICTED: fatty acid synthase-like [*Acyrthosiphon pisum*]**  **Swissprot: fatty acid synthase OS = *Rattus norvegicus* GN = Fasn PE = 1 SV = 3** | **7.56E-05** | **11.3365** |
|  | **Unigene27943_All^a^** | **gi\|189233593\|ref\|XP_970417.2\|** | **PREDICTED: similar to fatty acid synthase [*Tribolium castaneum*]**  **Swissprot: fatty acid synthase OS = *Mus musculus* GN = Fasn PE = 1 SV = 2** | **1.96E-10** | **5.3289** |
|  | **Unigene33063_All^a^** | **gi\|328721179\|ref\|XP_001949072.2\|** | **PREDICTED: fatty acid synthase-like [*Acyrthosiphon pisum*]**  **Swissprot: fatty acid synthase OS= *Homo sapiens* GN = FASN PE = 1 SV = 3** | **1.12E-11** | **3.5401** |
|  | **Unigene29675_All^a^** | **gi\|189233593\|ref\|XP_970417.2\|** | **PREDICTED: similar to fatty acid synthase [*Tribolium castaneum*]**  **Swissprot: fatty acid synthase OS = *Gallus gallus* GN = FASN PE = 1 SV = 5** | **7.84E-05** | **3.4733** |
|  | **Unigene32815_All^a^** | **gi\|307207457\|gb\|EFN85168.1\|** | **fatty acid synthase [*Harpegnathos saltator*]** | **3.20E-06** | **3.354** |
|  | **CL2044.Contig1_All^a^** | **gi\|345479260\|ref\|XP_001605059.2\|** | **PREDICTED: fatty acid synthase-like isoform 1 [*Nasonia vitripennis*]**  **Swissprot: fatty acid synthase OS = *Homo sapiens* GN = FASN PE = 1 SV = 3** | **1.32E-24** | **3.3461** |
|  | **Unigene24692_All^a^** | **gi\|328721179\|ref\|XP_001949072.2\|** | **PREDICTED: fatty acid synthase-like [*Acyrthosiphon pisum*]**  **Swissprot: fatty acid synthase OS = *Homo sapiens* GN = FASN PE = 1 SV = 3** | **5.83E-04** | **2.3438** |
| **acetyl coenzyme**  **A (CoA) carboxylase** | **Unigene22735_All^a^** | **gi|261599098|gb|ACX85645.1|** | **GH09643p [*Drosophila melanogaster*]**  **Swissprot: acetyl-CoA carboxylase 1 OS = *Rattus norvegicus* GN = Acaca PE = 1 SV = 1** | **5.51E-04** | **11.5333** |
|  | **Unigene33206_All^a^** | **gi\|328785413\|ref\|XP_624665.2\|** | **PREDICTED: acetyl-CoA carboxylase-like isoform 2 [*Apis mellifera*]**  **Swissprot: acetyl-CoA carboxylase OS = *Gallus gallus* GN = ACAC PE = 1 SV = 1** | **1.09E-08** | **5.0813** |
|  | **Unigene27368_All^a^** | **gi\|158292709\|ref\|XP_001688518.1\|** | **AGAP005175-PA [*Anopheles gambiae str*. PEST] >gi\|158292711\|ref\|XP_314071.4\| AGAP005175-PB [*Anopheles gambiae str.* PEST] >gi\|157017120\|gb\|EDO64101.1\| AGAP005175-PA [*Anopheles gambiae str.* PEST] >gi\|157017121\|gb\|EAA09449.5\| AGAP005175-PB [*Anopheles gambiae str.* PEST]**  **Swissprot: acetyl-CoA carboxylase 1 OS = *Mus musculus* GN = Acaca PE = 1 SV = 1** | **2.56E-08** | **4.1686** |
|  | **Unigene27811_All^a^** | **gi\|328714419\|ref\|XP_003245354.1\|** | **PREDICTED: acetyl-CoA carboxylase-like isoform 4 [*Acyrthosiphon pisum*]**  **Swissprot: acetyl-CoA carboxylase OS = *Gallus gallus* GN = ACAC PE = 1 SV = 1** | **4.65E-16** | **3.9084** |
|  | **Unigene10979_All^a^** | **gi\|158292709\|ref\|XP_001688518.1\|** | **AGAP005175-PA [*Anopheles gambiae str.* PEST] >gi\|158292711\|ref\|XP_314071.4\| AGAP005175-PB [*Anopheles gambiae str.* PEST] >gi\|157017120\|gb\|EDO64101.1\| AGAP005175-PA [*Anopheles gambiae str.* PEST] >gi\|157017121\|gb\|EAA09449.5\| AGAP005175-PB [*Anopheles gambiae str.* PEST]**  **Swissprot: acetyl-CoA carboxylase OS = *Gallus gallus* GN = ACAC PE = 1 SV = 1** | **2.85E-23** | **3.2873** |
|  | **Unigene20105_All^a^** | **gi\|189238375\|ref\|XP_969851.2\|** | **PREDICTED: similar to acetyl-coa carboxylase [*Tribolium castaneum*]**  **Swissprot: acetyl-CoA carboxylase 2 OS = *Homo sapiens* GN = ACACB PE = 1 SV = 3** | **7.45E-31** | **2.2598** |

**The corner marks of gene ID: ‘a’ represents up-regulated genes. The expression fold changes were performed with log 2 ratio and |log_2_Ratio| ≥ 2.**
